# Serum Metabolomics of Retinoblastoma: Assessing the Differential Serum Metabolic Signatures of Unilateral and Bilateral Patients

**DOI:** 10.1021/acsomega.3c07424

**Published:** 2023-12-07

**Authors:** Khushboo Gulati, Radhika Manukonda, Manikyaprabhu Kairamkonda, Swathi Kaliki, Krishna Mohan Poluri

**Affiliations:** † The Operation Eyesight Universal Institute for Eye Cancer, LV Prasad Eye Institute, Hyderabad-500034, Telangana, India; ‡ Brien Holden Eye Research Center, L. V. Prasad Eye Institute, Hyderabad-500034, Telangana, India; § Department of Biosciences and Bioengineering, Indian Institute of Technology Roorkee, Roorkee-247667, Uttarakhand, India; ∥ Centre for Nanotechnology, Indian Institute of Technology Roorkee, Roorkee-247667, Uttarakhand, India

## Abstract

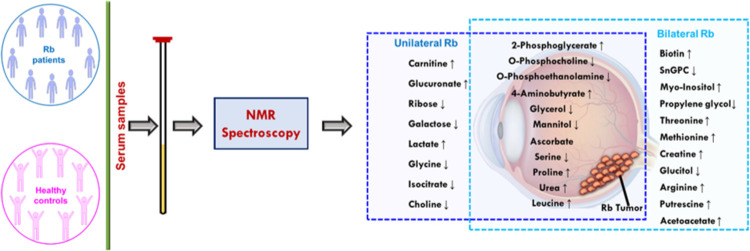

Retinoblastoma (Rb) is the most common pediatric eye cancer. To identify the biomarkers for early diagnosis and monitoring the progression of Rb in patients, mapping of the alterations in their metabolic profiles is essential. The present study aims at exploring the metabolic disparity in serum from Rb patients and controls using NMR-based metabolomics. A total of 72 metabolites, including carbohydrates, amino acids, and organic acids, were quantified in serum samples from 24 Rb patients and 26 controls. Distinct clusters of Rb patients and controls were obtained using the partial least-squares discriminant analysis (PLS-DA) model. Further, univariate and multivariate analyses of unilateral and bilateral Rb patients with respect to their age-matched controls depicted their distinct metabolic fingerprints. Metabolites including 2-phosphoglycerate, 4-aminobutyrate, proline, *O*-phosphocholine, *O*-phosphoethanolamine, and Sn-glycero-3-phosphocholine (Sn-GPC) showed significant perturbation in both unilateral and bilateral Rb patients. However, metabolic differences among the bilateral Rb cases were more pronounced than those in unilateral Rb cases with respect to controls. In addition to major discriminatory metabolites for Rb, unilateral and bilateral Rb cases showed specific metabolic changes, which might be the result of their differential genetic/somatic mutational backgrounds. This further suggests that the aberrant metabolic perturbation in bilateral patients signifies the severity of the disease in Rb patients. The present study demonstrated that identified serum metabolites have potential to serve as a noninvasive method for detection of Rb, discriminate bilateral from unilateral Rb patients, and aid in better understanding of the RB tumor biology.

## 1 Introduction

Retinoblastoma (Rb) is a pediatric eye cancer that originates as a result of mutations in the *RB1* gene, the first identified tumor suppressor gene.^1,2^ Each year, 5000–8000 cases of Rb are diagnosed worldwide. Rb constitutes 4% of all childhood cancer cases.^3^ Rb is observed in two differential clinical forms including the heritable and nonheritable forms.^4^ Heritable Rb cases are characterized by the bilateral and multifocal tumor at an early age. In contrast, nonheritable Rb cases are marked with unilateral and unifocal tumor, at a later age.^4^ Of all, 60% of Rb cases are unilateral, and not all of them are nonheritable. In contrast, 40% Rb cases are bilateral, and all of them are heritable. These unilateral and bilateral Rb cases are an outcome of nongerminal and germinal mutations of the *RB1* gene, respectively. This has been explained by Knudson who proposed two hit hypotheses in 1971, according to which Rb develops when there is loss or mutation of both the alleles of the *RB1* gene present on chromosome 13q1.4.^5^ In heritable Rb patients, the first hit refers to the inherited germline mutation in the *RB1* gene, and the second hit refers to the acquired secondary mutation during retinal development. Nonhereditary Rb patients acquire mutations in both the alleles of the *RB1* gene in the somatic cells of retina.^5,6^ Hereditary Rb patients are more susceptible to other malignancies including melanomas, soft tissue sarcomas, and osteosarcomas.^7^

The *RB1* gene encodes for the pRb protein which acts as a regulator in the cell-cycle check point during the G1/S transition.^8^ It has been marked that the pRb protein is not only essential for retinal cells for their appropriate cell-cycle exit, however, is also associated with several other metabolic activities via interaction with its binding partners in the cell.^9,10^ The functional loss of pRb and alterations in its associated metabolic pathways are majorly responsible for the aggressive Rb phenotype.^8,11−13^ Reprogramming of metabolic circuits is one of the major hall marks of cancer cells to satisfy their bioenergetic, biosynthetic, and redox demands.^14,15^ Dissecting the metabolic alterations reflects the phenotype of an organism and further aids in figuring out the metabolic processes associated with the disease.^16−18^ Therefore, it is quintessential to investigate the metabolic distortions associated with Rb to unravel the mechanisms that are underlying the Rb pathology.

Several research groups across the globe conducted metabolomic studies for retinoblastoma with the aim to understand and identify differential tumor staging for appropriate clinical decisions.^19−24^ These studies employed distinct sample types, including tissue, tear, vitreous humor, and aqueous humor. Although serum being an ideal and informative fluid that has been utilized for discerning the key metabolic alterations associated with several cancer types,^25−30^ it has not been scanned for retinoblastoma to date. Serum metabolomic profiling is an influential technique to investigate biomarkers for disease diagnostics/prognostics and also aided in proposing a suitable treatment strategy for different cancers including breast, lung, brain, gastric, and so on.^31−35^ Thus, the serum metabolic disparity among the Rb patients has been explored, which allows us to scan their metabolic aberrations as well as provides an opportunity to acquire mechanistic insights underlying Rb along with probing the potential diagnostic and prognostic markers for Rb.

To obtain comprehensive insights into Rb-related metabolomic changes, nuclear magnetic resonance (NMR) spectroscopy-based serum metabolomics has been employed. There are several added advantages of employing NMR spectroscopy for metabolomics, including its nondestructive nature (NMR samples can be reused for other assays), inherently quantitative, high reproducibility of outcomes, and minimal sample preparation steps.^36^ Thus, in the present study, detailed NMR-based serum metabolomics analysis was carried out in serum samples from Rb patients. Further, metabolomic aberrations among the unilateral and bilateral Rb patients were also erected. Results depicted the distinct serum metabolic profiles of Rb patients in contrast to controls. Interestingly, both unilateral and bilateral Rb patients shared a synonymous trend of the expression of discriminatory metabolites. However, the extent of such metabolic modulations is more pronounced in bilateral Rb patients in comparison with their unilateral counterpart. The current study also highlights the key metabolites including glucitol, myoinositol, acetoacetate, arginine, creatine, biotin, methionine, threonine, and biotin that exhibited significant alterations specifically in bilateral Rb patients, which can be further assessed for their biomarker potency.

## 2 Results

### 2.1 Patient Characteristics

**Figure 1:**
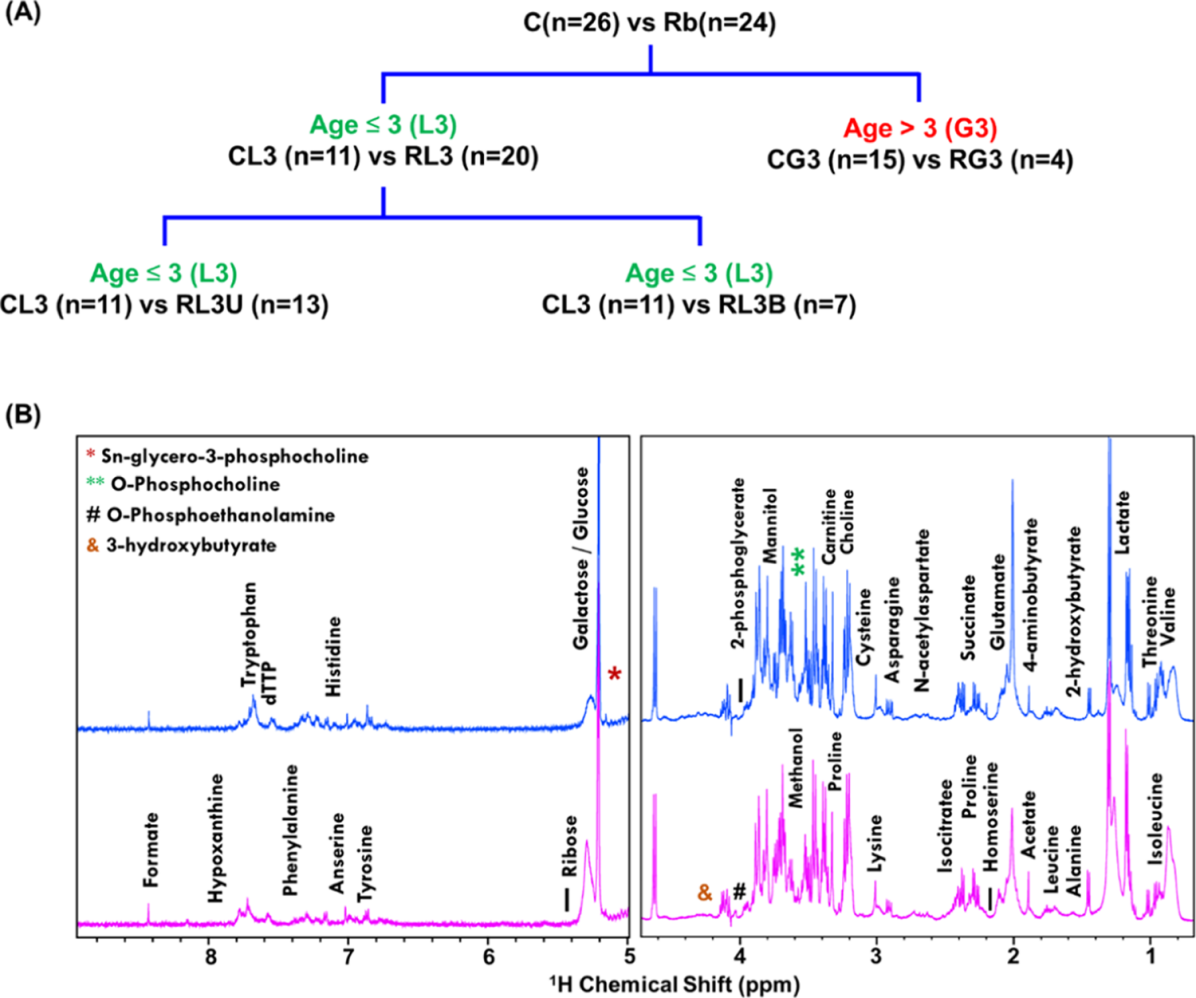
(A) Flowchart depicting the flow of analysis and number of serum samples from Rb patients and controls used in the designed study. C and Rb denote all controls and Rb patients; CL3 and CG3: controls ≤3 and >3 years of age, respectively; RL3 and RG3: Rb patients ≤3 years and >3 years of age, respectively; RL3U and RL3B: unilateral and bilateral Rb patients ≤3 years; RG3: Rb patients >3 years of age. All RG3 patients had unilateral Rb, thus represented as RG3U. n: number of serum samples. (B) Stack of one-dimensional (1D) ^1^H NMR Carr–Purcell–Meiboom–Gill (CPMG) spectra of controls (blue) and Rb patients (red). Assigned metabolites are colored red in the spectra. The water region at δ 4.5–4.9 was precluded for better resolution. The following abbreviations are used: Val: valine, Ileu: isoleucine, Thr: threonine, GABA: 4-aminobutyrate, Glu: glutamic acid, Asn: asparagine, Cys: cysteine, PC: *O*-phosphocholine, His: histidine, tyr: tyrosine, Phe: phenylalanine, and Trp: tryptophan.

The study involved serum samples from 24 Rb patients (Rb) and 26 controls (C). The demographic and clinical details of all of these subjects have been summarized in Table S1. Serum samples from both Rb patients and controls were segregated based on different age groups (Figure 1A). Out of 26 control samples, 11 were less than 3 years of age (CL3), and 15 were greater than 3 years of age (CG3). Out of 24 Rb patients, 20 were less than equal to 3 years (RL3), and 4 were greater than 3 years of age (RG3). Among Rb patients, two distinct forms of Rb cases were observed. The maximum number of Rb patients (*n* = 17) had unilateral intraocular Rb, in which 13 belonged to RL3U (less than 3 years of age with unilateral Rb) and 4 to RG3U (more than 3 years of age with unilateral Rb) category. Seven patients had bilateral intraocular Rb, and all of them belonged to the RL3B category (less than 3 years of age with bilateral Rb).

### 2.2 Decoding the Metabolic Alterations between Rb Patients and Controls

To elucidate the metabolic heterogeneity among the Rb patients with respect to controls, a comparative analysis of NMR-based serum metabolomics for all Rb patients (*n* = 24) with respect to all control samples (*n* = 26) has been performed. To further explore the metabolic alterations among the unilateral and bilateral Rb cases, metabolomics analysis for the age-matched Rb cases (for both unilateral and bilateral) with respect to age-matched controls was also performed as shown in the flowchart in Figure 1A.

#### 2.2.1 Metabolite Identification and Statistical Analysis

**Figure 2:**
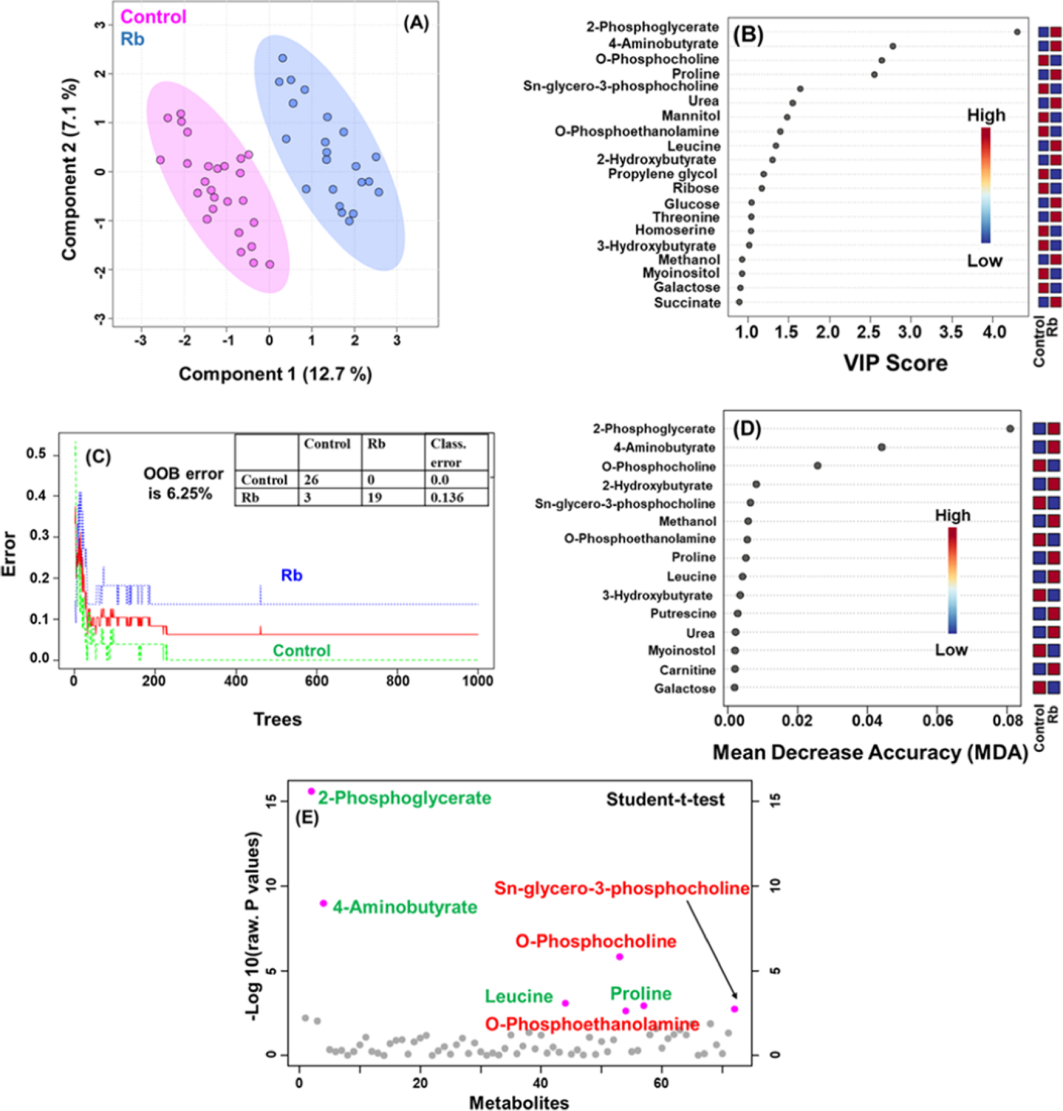
Multivariate statistical analysis of Rb patients wrt controls. (A) Representative partial least-squares discriminant analysis (PLS-DA) score plot obtained using the concentration data set of 72 metabolites assigned to 1D ^1^H NMR spectra of controls and Rb patients. The semitransparent ovals, pink (controls) and light blue (Rb), represent a 95% confidence interval. (B) PLS-DA-based variable of importance projection (VIP) score plot depicting the top 20 metabolites significantly discriminating the Rb patients group from the control group. Red and blue boxes in the right indicate the highest and lowest concentration of metabolites, respectively, in the control and Rb samples. (C) Random forest classification model depicting cumulative error rates measured for both groups (control and Rb patients) employing the RF machine learning approach. The overall error rate is indicated by the red line. Blue and green lines indicate the error rate for control and Rb groups, respectively. The out-of-bag error for the RF model generated was 6.25% as shown in the inset. (D) Mean decrease accuracy (MDA) score plot generated based on RF analysis when the features/metabolites were permuted, and their statistical significance was further evaluated using Student’s *t* test as shown in panel (E). Upregulated and downregulated metabolites are highlighted in green and red colors, respectively.

One-dimensional proton NMR spectral profiling has the capability to decode the diverse physiological patterns among the distinct serum samples and furnishes a metabolic snapshot with high resolution. Stack of ^1^H NMR spectra recorded for serum samples from Rb patients and controls is shown in Figure 1B. To perform multivariate analysis, peaks in the CPMG spectra were assigned to specific metabolites by utilizing the 500 MHz metabolite spectral database library contained in the Chenomx NMR software package and human metabolome database. A total of 72 metabolites annotated were categorized broadly into specific groups including amino acids and their derivatives (29), nucleic acids (2), carbohydrates (9), organic compounds (16), and others (16) (Table S2). To obtain an overview of the initial grouping trend and class separation, the normalized concentration data sets were analyzed using the unsupervised principal component analysis (PCA) method. The PCA score plots exhibited group clustering which implies discrimination between Rb patients and controls (Figure S1). Further, supervised partial least-squares discriminant analysis (PLS-DA) statistical analysis was carried out to obtain better separation between the Rb patients and controls (Figure 2A). A PLS-DA score plot clearly exhibited statistically significant discrimination between the two groups (Rb and control) as two distinct clusters were observed. PCA and PLS-DA models generated imply the metabolic variations among Rb patients in contrast to controls. The statistical significance of the PLS-DA model obtained was cross-validated using a 10-fold cross-validation algorithm, accuracy parameters, and permutation of the data set (Figure S2).

Variable importance on projection (VIP) score plot analysis was carried out to identify the most significant metabolites that are responsible for the group discrimination between the Rb and control groups. All of the metabolites with VIP scores greater than 1.0 are considered metabolites with high discriminatory potency to distinguish between the two groups. Top 16 metabolites including 2-phosphoglycerate, 4-aminobutyrate, *O*-phosphocholine, proline, Sn-glycerol-3-phosphocholine (Sn-GPC), urea, mannitol, *O*-phosphoethanolamine, leucine, 2-hydroxybutyrate, propylene glycol, ribose, glucose, threonine, homoserine, 3-hydroxybutyrate, methanol, and myoinositol were found to be significantly different in Rb patients in contrast to controls (Figure 2B). An advanced machine learning algorithm (Random Forest, RF) was used to further identify and confirm the discriminatory metabolites between the Rb and non-Rb cases. The RF algorithm evidenced differential serum metabolic profiles of Rb cases with respect to controls (Figure 2C). The out-of-bag error obtained was 6.25%, which implies a high prediction accuracy value of around 93.75%. Based on the Random Forest model, a mean decrease accuracy plot was generated which represents the significant discriminatory feature/metabolites between the groups. Higher mean decrease accuracy value indicates higher contribution of the metabolite toward discriminating the groups. Hence, MDA plot provides the imperative information related to metabolic alterations underlying the pathological state of a disease (Figure 2D). Around 15 metabolites showed significant MDA values (>0.01); of these 13 metabolites including 2-phosphoglycerate, 4-aminobutyrate, 2-phosphocholine, Sn-GPC, methanol, O-phosphoethanolamine, proline, leucine, 3-hydroxybutyrate, urea, and myoinositol were congruent with the VIP score plot analysis, indicating their significant contribution toward metabolic aberrations in Rb patients. The statistical significance of identified discriminatory metabolites was further assessed using Student’s *t* test, with a *p*-value threshold set to <0.05. Increased serum levels of 2-phosphoglycerate, 4-aminobutyrate, and leucine and decreased levels of *O*-phosphocholine, Sn-glycero-3-phosphocholine, proline, and *O*-phosphoethanolamine have been observed in Rb cases in contrast to controls (Figure 2E). Overall, the perturbed concentration of these metabolites differentiating the two groups (Rb patients and controls) clearly indicates the fluttered metabolic pathways in Rb patients.

**Figure 3:**
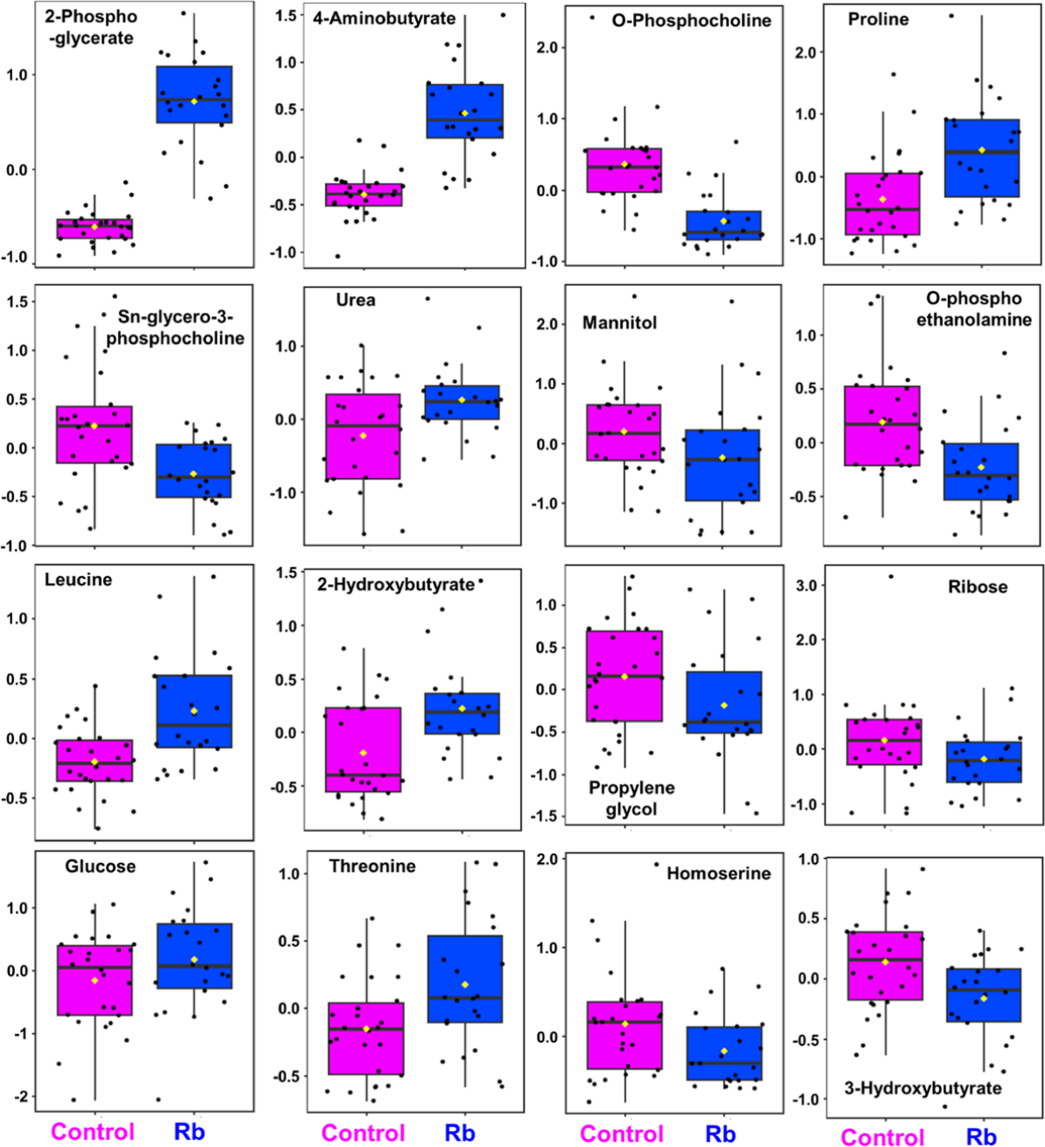
Representative box-cum-whisker plots of significant metabolites (with VIP score greater than 1.0) identified using VIP score plot generated on the basis of the PLS-DA model obtained for control versus Rb patients. The vertical axis represents the normalized concentration of metabolites. For the observed metabolic entities, the statistical significance was at the level of *p* ≤ 0.05. Plots depict the metabolic variation in Rb patients with respect to controls. In box plots, boxes represent the interquartile range, and the horizontal line inside the box indicates the median. Top and bottom border lines of the box represent the 75th and 25th percentiles, respectively. Upper and lower whiskers represent the 95th and 5th percentiles, respectively.

To further assess the differential expression of the discriminatory metabolites, box-cum-whisker plots of the top 16 distinct metabolites with relative normalized concentrations have been plotted (Figure 3). The plots clearly revealed the quantitative variation in relative signal integrals for the serum metabolites from Rb patients and controls. Box plots revealed the upregulation of 2-phosphoglycerate, 4-aminobutyrate, urea, proline, leucine, 2-hydroxybutyrate, glucose, and threonine. In contrast, the metabolites including *O*-phosphocholine, *O*-phosphoethanolamine, Sn-glycero-3-phosphocholine, mannitol, propylene glycol, ribose, homoserine, and 3-hydroxybutyrate were downregulated in Rb patients as compared to controls.

Fold change (FC) of significantly distinct metabolites in Rb patients was assessed on the basis of variation in the FC threshold of ≥1.4 in comparison to controls. A total of 6 metabolites were identified that exhibited FC ≥ 1.4 in Rb patients with respect to controls (Table 1). Metabolites exhibiting significantly lower levels in Rb cases include *O*-phosphocholine, Sn-glycero-3-phosphocholine, and *O*-phosphoethanolamine. In contrast, significantly elevated levels of 2-phosphoglycerate, 4-aminobutyrate, and proline were found in Rb samples. The global overview of significantly altered metabolites among Rb patients and controls has also been depicted by the heat map constructed for the top 25 significantly discriminatory metabolites (Figure S3). These results clearly established that metabolic profiles of Rb patients are remarkably different from those of controls in terms of differential expression patterns of metabolites. Among the identified metabolites, a relatively high concentration of 15 metabolites was observed in Rb patients, whereas reduced levels of 10 metabolites were marked in serum from Rb patients with respect to controls. Metabolites including proline, leucine, 4-aminobutyrate, threonine, and taurine showed contrastingly lower levels in serum from controls than from Rb patients’ group (Figure S3).

**Table 1:** List of Metabolites Significantly Altered in Rb Patients with Respect to Controls^a^

s. no.	metabolite name	fold change	Log2(FC)
1	*O*-phosphocholine	0.34	–1.54
2	2-phosphoglycerate	2.62	1.39
3	4-aminobutyrate	2.36	1.24
4	proline	1.81	0.86
5	sn-glycero-3-phosphocholine	0.63	–0.65
6	*O*-phosphoethanolamine	0.63	–0.64

a The fold change threshold value 1.4 was considered and log2(FC) values, possessing *p*-value <0.05. The fold change values >1 and <1 imply the upregulation and downregulation of the metabolites, respectively.

#### 2.2.2 Assessing the Altered Metabolic Pathways in Rb Patients

**Figure 4:**
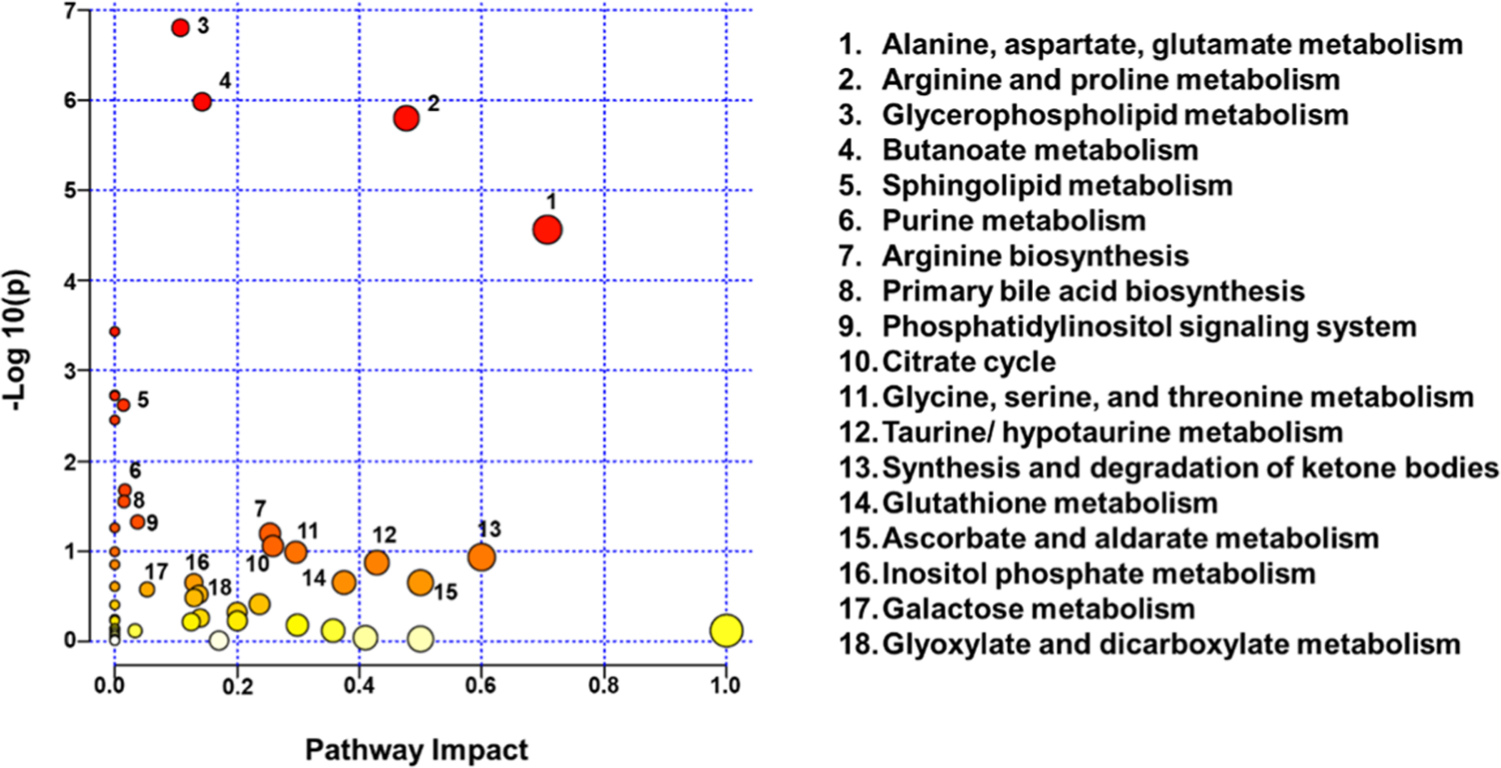
Metabolic pathway impact plot showing the altered pathways as an outcome of altered metabolic activities in the Rb patient group as compared to controls. The *x*-axis depicts the pathway impact value based on the pathway topological analysis, and the *y*-axis implies the −log10(*p*) value computed on the basis of pathway enrichment analysis. Node color gradient from red to yellow indicates the pathway enrichment score. Red and yellow colors imply the higher and lower values of −log10(*p*), respectively. The size of the circle is directly proportional to the pathway impact value. Bigger and smaller circles represent the higher and lower impact values, respectively.

Pairwise pathway impact analysis was conducted to underpin the pathways perturbed under Rb conditions. The concentration data set of all 72 metabolites from the serum samples of both Rb patients and control groups was utilized for the pathway impact analysis that showed perturbation in 49 different metabolic pathways. The metabolic pathways with −log10(*p*) ≥ 0.5 and impact value >0.01 were selected as the most perturbed metabolic pathways in Rb patients in contrast to controls (Table S3). Calculations of pathway impact values were based on the topological analysis and those of −log10(*p*) were based on enrichment analysis. Eighteen metabolic pathways exhibited −log10(*p*) values ranging from 0.5 to 6.8 and impact value >0.01 (Figure 4). The size/area of the circle is in direct correlation with the pathway impact. The color of the circle implies the level of significance; red and white colors imply the highest and lowest significance values, respectively. These highly enriched pathways mainly belong to the carbohydrate, amino acid, and energy metabolism. One of the most significant pathways with the highest impact value (0.71) is the alanine, aspartate, and glutamate metabolism pathway (Figure 4). This pathway is also highly enriched with perturbed levels of metabolites including *N*-acetyl-l-aspartate, alanine, citrate, aspartate, glutamate, pyruvate, glutamine, 4-aminobutyrate, and succinate, of which *N*-acetyl-l-aspartate, 4-aminobutyrate, and succinate showed maximum modulation in Rb patients.

Other remarkable pathways along with key metabolites that have been altered in Rb patients include the following (Figure 4): (a) arginine and proline metabolism (proline, 4-aminobutyrate, putrescine); (b) glycerophospholipid metabolism (phosphocholine, Sn-glycero-3-phosphocholine); (c) butanoate metabolism (4-aminobutyrate, 3-hydroxybutyrate); (d) sphingolipid metabolism (phosphoethanolamine); (e) purine metabolism (glutamine, hypoxanthine); (f) arginine biosynthesis (l-aspartate, l-arginine, urea); (g) primary bile acid biosynthesis (taurine); (h) phosphatidylinositol signaling system (myoinositol); (i) citrate cycle (pyruvate, malate, succinate, citrate, isocitrate); (j) glycine, serine, and threonine metabolism (l-ornithine, putrescine, l-glycine); (k) taurine/hypotaurine metabolism (taurine); (l) synthesis and degradation of ketone bodies (3-hydroxybutanoate); (m) ascorbate and aldarate metabolism (myoinositol, glucuronate); (n) inositol phosphate metabolism (myoinositol, d-glucuronate); (o) galactose metabolism (myoinositol, d-galactose); and (p) glyoxylate and dicarboxylate metabolism (citrate, isocitrate, pyruvate, glycine). Alteration of these metabolic pathways is an outcome of differential gene expression patterns of their associated molecular partners in Rb patients. The significance of these varied signatory metabolites in Rb has been discussed in later sections.

### 2.3 Dissecting the Metabolic Disparity between Unilateral and Bilateral Rb Patients with Age-Matched Controls

The observed metabolic alterations between the Rb and control samples warrant a thorough analysis of the factors contributing to these perturbations, as differential regulation can be the result of various physiological/environmental factors. Among the factors that influence the metabolic profiles, two striking factors are (a) age of the children and (b) unilateral vs bilateral Rb. The following subsections provide fine details of metabolic perturbations due to these genetic/dietary influences.

#### 2.3.1 Differential Metabolic Features of Control Samples with Varied Child Age

The age of the children plays a primary role in comparative metabolomics of Rb patients, as the metabolic profile of serum is dependent on the dietary effects and physical activities that vary greatly between the children of age groups 1–3 years and above 3 years.^37−41^ A comparative analysis between CL3 (controls less than 3 years) and CG3 (controls greater than 3 years) was carried out to discern the metabolic differences among the controls owing to their differential age groups. PLS-DA showed distinct clusters for CL3 and CG3 with cross-validation values including accuracy = 0.86, *R*^2^ value = 0.64, and *Q*^2^ value = 0.35 (Figure S4). The top discriminatory metabolites with VIP score >1 distinguishing the CL3 from CG3 groups are shown in Figure S4C. This clearly indicates the differential metabolic profiles among the controls based on their ages, implying the heterogeneity among the serum samples from controls. Hence, to rectify the interference of the age-based metabolic variations, further metabolic analysis of unilateral/bilateral Rb patients was performed with their age-matched controls to avoid the confounding outcomes.

#### 2.3.2 Differential Metabolic Features of Age-Matched Unilateral and Bilateral Rb Patients

**Figure 5:**
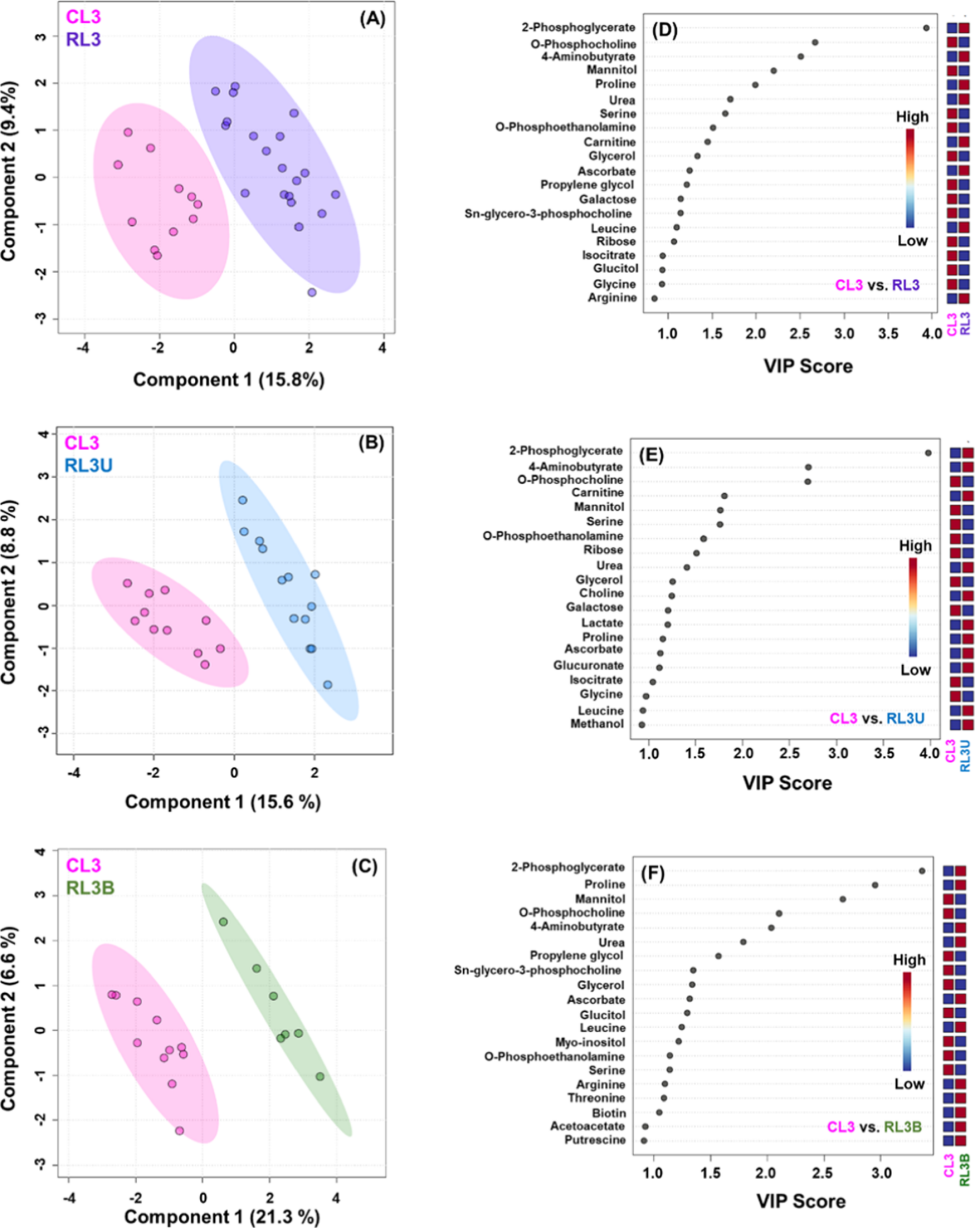
Representative 2D PLS-DA score plots generated for (A) CL3 versus RL3, (C) CL3 versus RL3U, and (E) CL3 versus RL3B, using pairwise PLS-DA of concentration data set of 72 metabolites annotated in the 1D ^1^H NMR spectra acquired for CL3 and RL3 subgroups (both RL3U and RL3B cases). Semitransparent ovals exhibit a 95% confidence interval. VIP score plots were obtained for (B) CL3 versus RL3, (D) CL3 versus RL3U, and (F) CL3 versus RL3B based on their corresponding pairwise PLS-DA subgroup analysis. VIP score plots depict the top 20 metabolites significantly altered among differential subgroups.

Multivariate statistical analysis was carried out to assess the serum metabolic profiles of RL3 (Rb patients ≤3 years) and RL3U/RL3B (Rb patients ≤3 years with unilateral/bilateral Rb) with respect to their age-matched controls CL3 (controls less than 3 years). PLS-DA models generated for CL3 vs RL3, CL3 vs RL3U, and CL3 vs RL3B marked an overall variation of 15.8, 15.6, and 21.3%, respectively (Figures 5A–C, S5). PLS-DA models generated for distinct subgroups clearly revealed that the extent of serum metabolic differences is higher in bilateral Rb cases (RL3B) than in unilateral Rb cases (RL3U) with respect to controls (CL3).

Further, VIP score plots were generated on the basis of PLS-DA models obtained for differential subgroups including CL3 vs RL3, CL3 vs RL3U, and CL3 vs RL3B (Figure 5D–F, Table S4). It has been observed that some of the top discriminatory metabolites (with VIP score ≥1) responsible for the discrimination between the different subgroups [RL3 vs CL3, RL3U vs CL3U, and RL3B vs CL3] were congruent. Such common discriminatory signatures include 2-phosphoglycerate, *O*-phosphocholine, 4-aminobutyrate (GABA), proline, *O*-phosphoethanolamine, urea, leucine, ascorbate, glycerol, serine, and mannitol (Figure 5D–F), Table S4. This implies that these are top signature metabolites which majorly discriminate the Rb patients from controls. In contrast, metabolites including carnitine, isocitrate, glycine, ribose, and galactose showed significant variations in RL3 vs CL3 and RL3U vs CL3. Such metabolic alterations in RL3 vs CL3 are due to the significant variations of these metabolites in unilateral Rb cases. Lactate, glucuronate, and choline showed significant variation in CL3 vs RL3U which implies that perturbation of these metabolites is a feature of unilateral Rb patients (Figure 5D–F, Table S4). Further, metabolites exhibited significant variation in RL3 vs CL3 and RL3B vs CL3. This implies that the significance of Sn-glycero-3-phosphocholine, propylene glycol, arginine, and glucitol in RL3 vs CL3 subgroup analysis is majorly contributed by significant alterations of these metabolites in bilateral Rb patients. Furthermore, threonine, myoinositol, biotin, acetoacetate, and putrescine exhibited significant alteration exclusively in bilateral Rb patients (Figure 5D–F, Table S4). Altogether, such detailed subgroup analysis helped in dissecting the hidden significant metabolic details specific to unilateral and bilateral Rb patients.

Additionally, fold change analysis for distinct subgroups including RL3 vs CL3, RL3U vs CL3U, and RL3B vs CL3 was also performed to identify significant metabolites with the fold change threshold value ≥1.4, with *p*-values <0.05 (Table 2). Metabolites including 2-phosphoglycerate and 4-aminobutyrate were observed as upregulated, and *O*-phosphoethanolamine, *O*-phosphocholine, and serine were found to be downregulated among all of the pairwise subgroup analysis. This implies the importance of these metabolites in discriminating Rb patients from controls, which is congruent with our VIP score plot analysis. Further, lactate, carnitine, and alanine with fold change values of around 1.67, 1.59, and 1.40 were uniquely upregulated in RL3U. In contrast, metabolites such as proline, putrescine, urea, mannitol, ascorbate, creatine, Sn-glycero-3-phosphocholine, myoinositol, leucine, acetoacetate, arginine, methionine, threonine, propylene glycol, glycerol, and biotin showed modulatory effects specifically in RL3B. Proline, biotin, ascorbate, and putrescine with FC values equal to 2.43, 1.71, 1.66, and 1.65, respectively, were remarkably increased in RL3B. In contrast, mannitol, Sn-glycero-3-phosphocholine, myoinositol, and glycerol with FC values of 0.42, 0.63, 0.63, and 0.70 showed significant depletion in serum samples from bilateral Rb patients (RL3B) (Table 2).

**Table 2:** List of Metabolites Significantly Altered in Different Rb Subgroups with Respect to Controls Including CL3 vs RL3, CL3 vs RL3U, CL3 vs RL3B^a^

		relative fold change		
s. no	metabolite name	CL3 vs RL3	CL3 vs RL3U	CL3 vs RL3B
1	*O*-phosphocholine	0.27	0.26	0.29
2	2-phosphoglycerate	2.92	2.89	2.98
3	4-aminobutyrate	2.59	2.50	2.75
4	*O*-phosphoethanolamine	0.56	0.54	0.58
5	serine	0.60	0.57	0.65
6	lactate	1.43	1.67	
7	carnitine	1.47	1.59	
8	alanine		1.40	
9	proline	1.69		2.43
10	putrescine	1.45		1.65
11	urea	1.40		1.63
12	mannitol	0.61		0.42
13	ascorbate	1.48		1.66
14	creatine			1.63
15	Sn-glycero-3-phosphocholine-		0.63	
16	myoinositol			0.63
17	leucine			1.56
18	acetoacetate			1.55
19	arginine			1.47
20	methionine			1.47
21	threonine			1.45
22	propylene glycol			0.70
23	glycerol			0.70
24	biotin			1.71

a The fold change threshold value 1.4 and log2(FC) values possessing *p*-value <0.05 were considered. The fold change values >1 and <1 imply the upregulation and downregulation of the metabolites, respectively.

**Figure 6:**
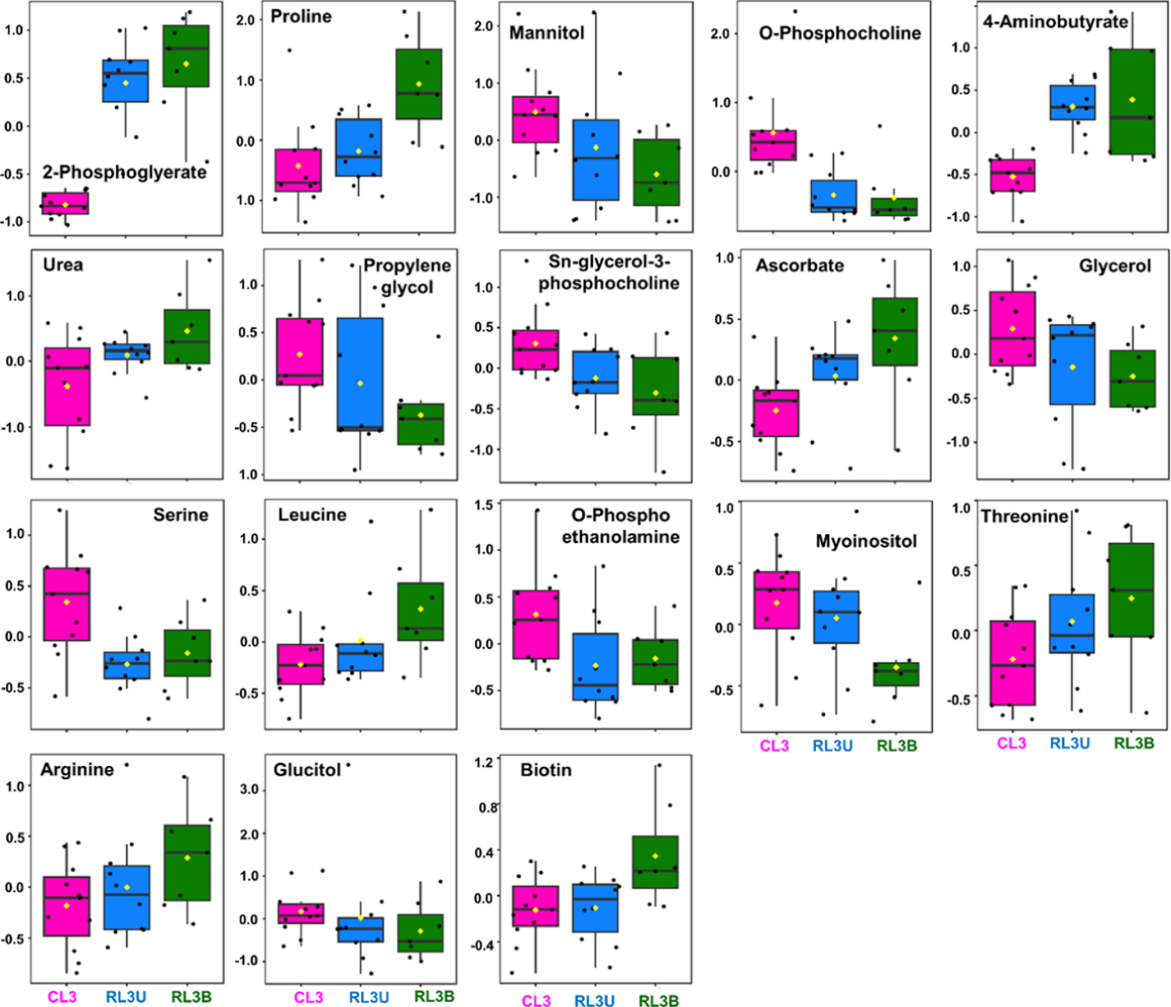
Box-cum-whisker plots of significant metabolites identified using the VIP score plot generated on the basis of the PLS-DA model obtained for CL3 versus RL3U and RL3B. The plots exhibit the altered metabolites in different Rb subgroups (RL3U, RL3B) with respect to CL3. The vertical axis represents the normalized concentration of metabolites. For the observed metabolic entities, the statistical significance is at the level of *p* ≤ 0.05. In box plots, boxes represent the interquartile range, and the horizontal line inside the box indicates the median. The top and bottom border lines of the box represent the 75th and 25th percentiles, respectively. The upper and lower whiskers exhibit the 95th and 5th percentiles, respectively.

An overview of notably distinct metabolites among the unilateral and bilateral Rb patients has also been illustrated by the heat map constructed for subgroups including RL3 vs CL3, RL3U vs CL3U, and RL3B vs CL3 (Figures S6–S8). The comparative analysis of heat maps clearly reveals relatively more metabolic variations in unilateral/bilateral Rb patients. Whisker-cum-box plots were generated to depict the patterns of upregulation/downregulation among the differential Rb subgroups (RL3U and RL3B) with respect to controls (CL3) (Figure 6). These box plots were generated on the basis of the PLS-DA model obtained by comparing 3 groups including CL3, RL3U, and RL3B, which showed a clear distinction between the groups, as all three groups evidenced distinguished clusters, thus depicting a variation of 16.8% (Figure S9). The box plot analysis illustrates that the metabolites including 2-phosphoglycerate, 4-aminobutyrate, ascorbate, proline, urea, leucine, threonine, arginine, and biotin showed an upregulation in both RL3U and RL3B as compared to CL3. However, the extent of upregulation is higher in RL3B in contrast to RL3U. Further, metabolites including mannitol, glycerol, *O*-phosphocholine, propylene glycol, *O*-phosphoethanolamine, myoinositol, serine, glucitol, and sn-3-glycero-3-phosphocholine showed downregulation in Rb cases (RL3U and RL3B) in contrast to controls (CL3). Similarly, the extent of downregulation is more in RL3B cases than in RL3U cases except serine for which the RL3U cases showed more downregulation than RL3B (Figure 6).

Furthermore, serum metabolic profiles of RG3 were compared with CG3. It should be noted that all of the samples in RG3 were found to be unilateral Rb cases. There was no sample for the bilateral Rb case in the RG3 group, since the possibility of development of bilateral Rb in children greater than 3 years of age is rare. Hence, the PLS-DA model was generated for the RG3U versus CG3 group only. The model showed a clear distinction between the two groups since both the groups exhibited distinguished clusters, depicting the variation of 15.7% (Figure S10A). The VIP score plot generated on the basis of the PLS-DA model depicted 16 important discriminatory metabolites (Figure S10B). Major metabolites include 2-phosphoglycerate, proline, 4-aminobutyrate, propylene glycol, *O*-phosphocholine, leucine, 2-hydroxybutyrate, fructose, Sn-glycero-3-phosphocholine, myoinositol, glycerol, glutamine, arginine, threonine, glucose, and ethanol (Figure S10B). It is worth noting that only a few discriminatory metabolites including 2-phosphoglycerate, 4-aminobutyrate, *O*-phosphocholine, leucine, and glycerol are common among RG3 and RL3U. The rest of the discriminatory metabolites are significantly distinct in RG3 as compared to RL3U. This implies that there do exist differences in the metabolic profiles of unilateral Rb patients belonging to distinct age groups. Hence, it is quintessential to consider the age factor while performing metabolomics analysis of Rb patients. The observed patterns need to be reconfirmed in future studies with a large data set of unilateral Rb patients above 3 years of age, to obtain affirmative conclusions on metabolic alterations of RL3U/RG3 patients.

#### 2.3.3 Differential Metabolic Pathways of Unilateral and Bilateral Rb Patients

**Figure 7:**
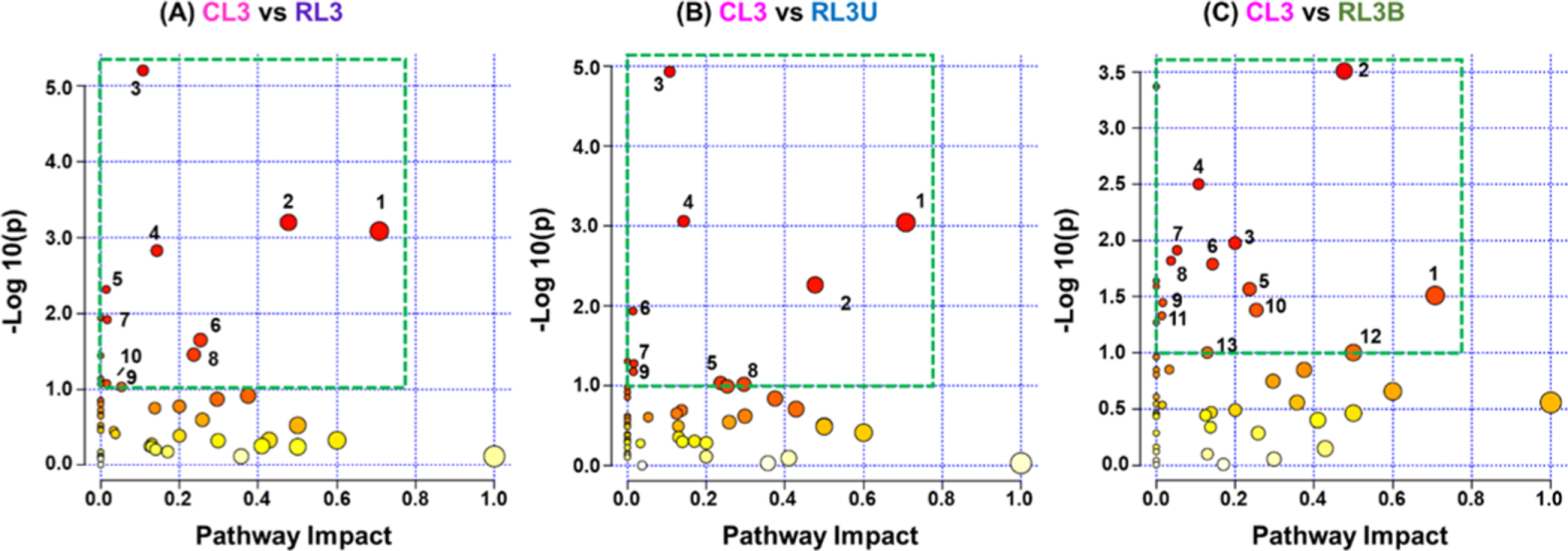
Metabolic pathway impact plots showing altered pathways in (A) CL3 vs RL3, (B) CL3 vs RL3U, and (C) CL3 versus RL3B. The *x*-axis exhibits the pathway impact values evaluated on the basis of pathway topological analysis, and the *y*-axis represents the −log10 of the *p*-value calculated based upon the pathway enrichment analysis. The metabolic pathways (in A, B, C) with −log(10)*p* value greater than 1.0 and impact value greater than 0.01 include alanine, aspartate, and glutamate metabolism (1, 1, 1), arginine and proline metabolism (2, 2, 2), glycerophospholipid metabolism (3, 3, 4), butanoate metabolism (4, 4, 6), sphingolipid metabolism (5, 6, 11), arginine biosynthesis (6, -, 10), purine metabolism (7, 7, 9), glycerolipid metabolism (8, 5, 5), galactose metabolism (9, -, 7), primary bile acid biosynthesis (10, 9, -), glycine, serine, and threonine metabolism (-, 8, -), biotin metabolism (-, -, 3), ascorbate and aldarate metabolism (-, -, 12), phosphatidylinositol signaling system (-, -, 8), and inositol phosphate metabolism (-, -, 13).

Pairwise pathway impact analysis was performed for different subgroups, CL3 vs RL3, CL3 vs RL3U, and CL3 vs RL3B, to identify the pathways being affected in unilateral and bilateral Rb patients (Figure 7). Overall, 50 metabolic pathways have shown perturbation among each subgroup (Tables S5–S7). The significantly perturbed pathways (with −log10(*p*) value >1.0 and impact value >0.01) are ranked which is directly related to metabolic fold changes and enrichment of specific pathways. Seven significantly perturbed metabolic pathways have been observed among each subgroup, of which glycerophospholipid metabolism, arginine and proline metabolism, alanine, aspartate, and glutamate metabolism are essentially more significant with higher −log10(*p*) values and pathway impact values among each subgroup (Figure 7A–C, Table S8). This indicates that the alteration of these metabolic pathways is a signature of Rb patients. The pathway impact analysis of CL3 versus RL3U also exhibited specific modulation in additional pathways related to primary bile acid synthesis and glycine, serine, and threonine metabolism (Figure 7B). Interestingly, six additional metabolic pathways including arginine biosynthesis, galactose metabolism, biotin metabolism, phosphatidylinositol signaling system, ascorbate and aldarate metabolism, and inositol phosphate metabolism reflected remarkable perturbation in RL3B. This observation highlights the pronounced metabolic variations associated with bilateral Rb patients (Figure 7C).

This also coincides with the variances observed in PLS-DA and the number of significant metabolites attained in fold change analysis for bilateral Rb patients in contrast to unilateral Rb patients. Both fold change and multivariate statistical analyses showed significant variations in both unilateral/bilateral Rb patients with respect to controls. However, the extent of alterations observed were more intense in the case of bilateral Rb patients as compared to their unilateral counterparts. The pathway impact analysis was in line with the multivariate analysis depicting the high enrichment (−log10(*p*) value >1.0) of metabolic pathways in bilateral Rb patients which is an outcome of their highly distorted metabolic system.

## 3 Discussion

### 3.1 Mechanistic Insight into Metabolomic Perturbations of Rb Patients

Rb, like other cancer types, is a heterogeneous disease.^42^ As a consequence of uncontrolled cellular growth, Rb cells require additional nutrients. To accomplish that, Rb cells reprogram their metabolic circuits.^19,43^ Since metabolic features of cancer cells are distinct from normal cells, metabolic pathways are being employed for tailoring cancer drugs.^44,45^ Moreover, alterations in serum metabolites could be potential biomarkers in the diagnosis and prognosis of Rb. Previous metabolomics-based studies made efforts to get insights into the Rb pathogenesis and classify the tumor to direct the treatment decisions at diagnostic levels.^19−22,24^ For instance, Kohe et al. diagnosed three distinctive metabolic subgroups of Rb based on the presence of three major prejudicial metabolites including taurine, hypotaurine, total choline, and creatine in tumor tissues.^21^ The same group investigated the metabolic differences between three pediatric tumors including medulloblastoma, retinoblastoma, and neuroblastoma and found that all three tumors exhibited the metabolic features of neural tissues.^22^ Sahoo et al. explored the definite differences between the healthy retina and Rb from the perspectives of metabolism/biochemical pathways by exploiting mathematical modeling strategies.^24^ On similar lines, Guha et al. elucidated transcriptomics and metabolomic profiles of Rb patients using different sample types including tear, vitreous humor, aqueous humor, and tissues.^20^

**Figure 8:**
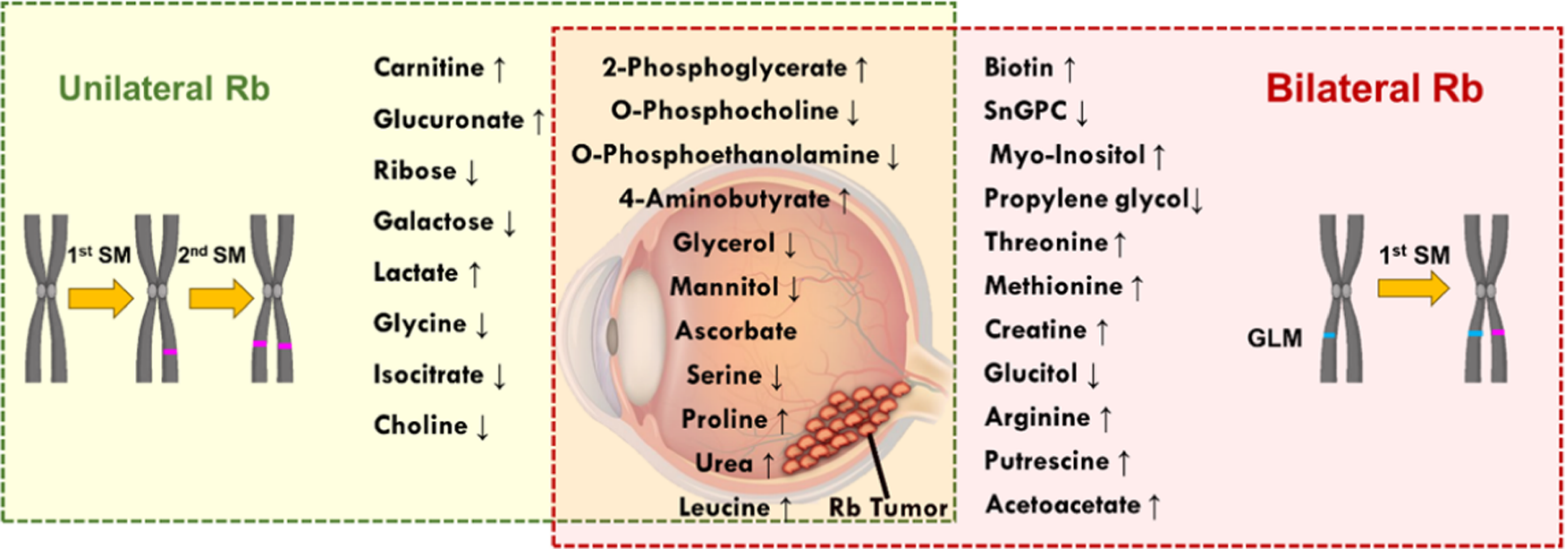
Schematic depicting the up/downregulation of metabolites that are common in Rb patients and specific to unilateral/bilateral Rb patients. SM: somatic mutation, GLM: germline mutation, and Sn-GPC: Sn-glycero-3-phosphocholine.

**Figure 9:**
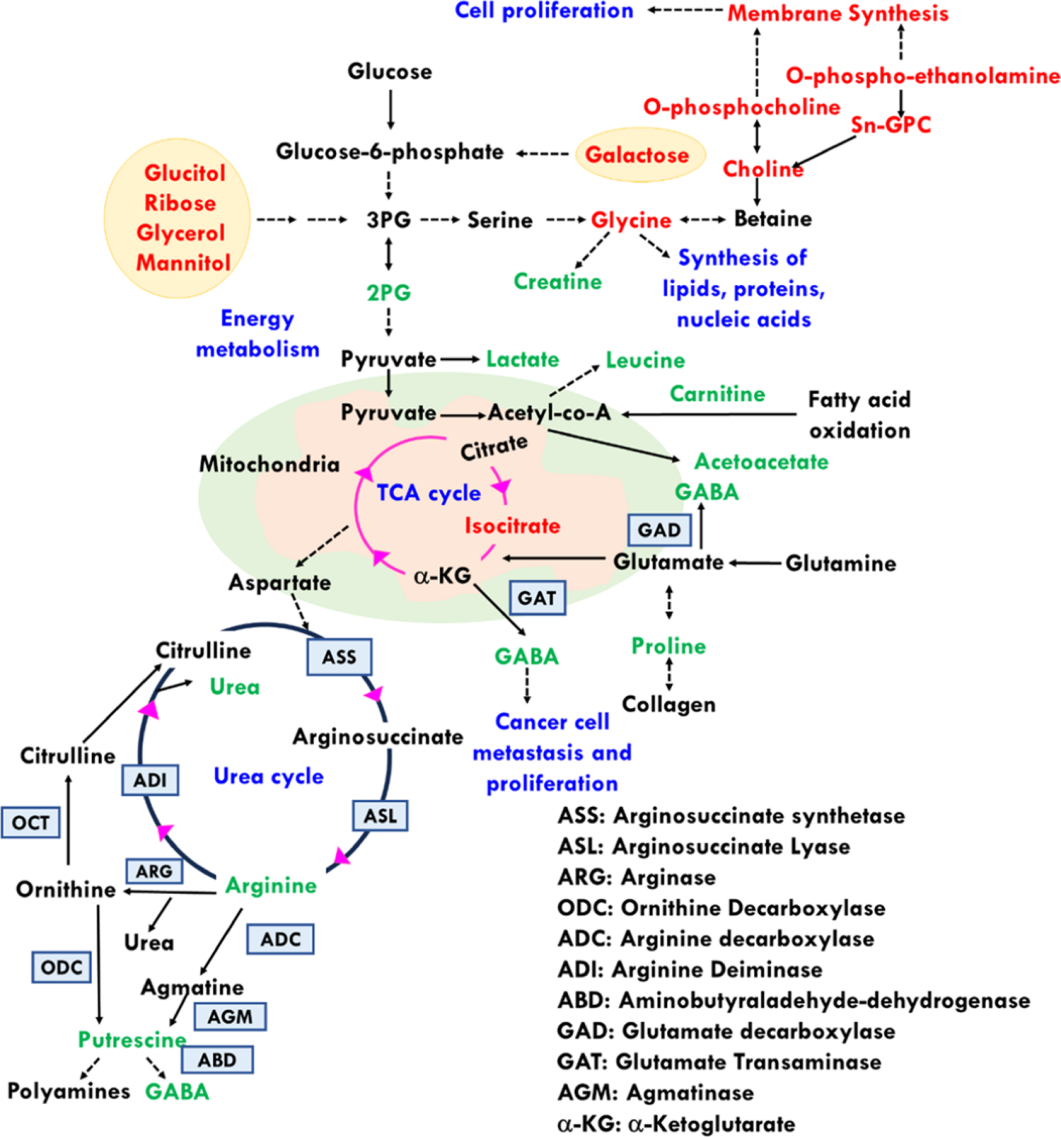
Schematic showing all of the perturbed metabolites along with their metabolic pathways. Up/downregulated metabolites are highlighted in green and red colors, respectively.

However, the assessment of serum metabolic profiles of Rb patients is still unscanned. The present study attempted to identify the similarities and differences in metabolites from the serum of Rb patients. Additionally, metabolic fingerprints specific to unilateral and bilateral Rb patients were also elucidated. A wide variety of metabolites, including energy metabolites, membrane metabolites, and amino acids, were observed in the serum from Rb patients and controls. Both univariate and multivariate analyses revealed the significant alteration of 6 major metabolites in all of the Rb patients’ groups despite their different age groups and laterality of the disease. Such metabolites including O-phosphocholine (PC), *O*-phosphoethanolamine (PE), 2-phosphoglycerate, 4-aminobutyric acid, proline, and sn-glycero-3-phosphocholine could be used and have potential to discriminate Rb patients from controls. Further, the age-based subgroup analysis revealed 11 common metabolites perturbed among both unilateral and bilateral Rb patients. However, the extent of their perturbation is higher in the case of patients suffering from bilateral Rb. Eight metabolites have shown disturbed levels specifically in unilateral Rb patients. In contrast, 11 metabolites were specially up/downregulated in bilateral Rb patients (Figure 8). Such specific metabolic changes might be the outcomes of their differential genetic/somatic mutational backgrounds. The implication of these metabolic changes in the pathophysiology of Rb has been discussed further (Figure 9).

An important feature discriminating the Rb patients from controls is the reduced levels of *O*-phosphocholine, *O*-phosphoethanolamine, and Sn-GPC in Rb indicating the modulation of the glycerophospholipid metabolic pathway in Rb patients. Alterations in phospholipids is associated with pathophysiology of several cancers. Increased levels of phosphatidylcholine (PtdC) and phosphatidylethanolamine (PtdE) have been observed in many cancer cells as they are involved in synthesis of building blocks of cells, thereby assisting in cancer cell proliferation.^46,47^ Based on these reports, we summarize that lower levels of PE and PC in serum of Rb patients are due to their significant conversion into PtdE and PtdC to meet the cell membrane requirements of Rb cells for their proliferation. Lower levels of sn-glycero-3-phosphocholine (Sn-GPC) indicate that it is being utilized by the cells to synthesize choline since Sn-GPC is a common precursor in the biosynthesis of choline.^48^ Hence, Sn-GPC might be replenishing the choline that is being rapidly used up by the Rb cells.

Depletion of differential sugar-related metabolites including glycerol and mannitol implies that these sugars metabolize to produce glucose, which enters into glycolysis cycle to fulfill the energy requirements of the rapidly proliferating cells. However, an increase in 2-phosphoglycerate levels (a glycolysis pathway intermediate) has been observed which implies that glucose is entering into glycolysis; however, it is not undergoing complete glycolysis (Tables 1 and 2). This implies a profoundly dampened glycolysis pathway. This is in line with the fact that Rb cells lacking functional Rb gene mediate a metabolic switch from glycolysis to oxidative phosphorylation (OXPHOS)-dependent adenosine triphosphate (ATP) generation.^43^ Interestingly, an increase in the lactate concentration was observed specifically in unilateral Rb patients. Lactate is produced as a byproduct of glycolysis in anaerobic conditions; however, it has been marked that in several cancers, lactate is also produced from glycolysis even in the presence of oxygen, which is termed as the Warburg effect.^49,50^ Our observations suggested that the glycolytic pathway is still active in unilateral Rb patients, accounting for their increased serum lactate levels. Increased serum levels of lactate have also been observed in gliomas, head and neck cancer, and breast cancer.^51−55^ Lactate is known as a fuel for oxidative metabolism in cancer cells^56−60^ and acts as a signaling mediator between cancer cells and endothelial cells, which mediates tumor growth and development.^60^ It acts as a major fuel for TCA cycle than glucose in lung and pancreatic cancers.^50^ Lactate has also been marked as the highest concentration metabolite in retinal tissues and in Rb tumor tissues.^22,61^ Reduced levels of the TCA cycle intermediate, isocitrate, further imply the utilization of the TCA cycle by Rb cells to meet their energy needs. Further, specific depletion of galactose and ribose in unilateral Rb patients also implies the utilization of these metabolites as alternate energy sources. Glioblastoma cells have been marked to metabolize galactose through the Leloir pathway for ATP production.^62^ Recently, it has been demonstrated that ribose acts as a fuel for the growth of pancreatic cancer cells.^63^ Altogether, these data imply the possibility of differential regulation of glycolytic and oxidative phosphorylation pathways by unilateral and bilateral Rb patients.

Increased levels of leucine, an essential branched chain amino acid, were observed in Rb. Increased intracellular levels of leucine have also been observed in other cancer types, and leucine is provided to cancer cells from tumor microenvironment or via breakdown of proteins.^64−66^ Leucine is involved in tumor development and progression. It acts as a building block for protein synthesis and also serves as a direct source of nitrogen for nucleotide biosynthesis via glutamate–glutamine axis.^67^ Increased levels of carnitine and proline were also observed in Rb patients. Carnitine serves as a shuttle molecule that carries fatty acid into mitochondria for their subsequent degradation by oxidation.^68^ Proline is known to play a crucial role in cancer, owing to its involvement in synthesis of collagen, a major constituent of extracellular matrix in tumor microenvironment.^69−71^ Meanwhile, lower levels of serine/glycine indicate their utilization to synthesize precursors required to produce additional lipids, proteins, and nucleic acids needed for the expanding biomass via one carbon metabolism.^72^

Increased levels of 4-aminobutyrate (GABA) have been observed in Rb patients in contrast to controls which is consistent with previous Rb studies.^22,24^ GABA is an imperative neurotransmitter in retina, conferring neurotrophic effects, thereby aiding in maturation of retina and its subsequent development.^73,74^ It is interesting to note the involvement of GABA in cancer cell metastasis and proliferation.^75^ GABA biosynthesis occurs via multiple routes: (1) involving the conversion of glutamate to GABA via glutamate decarboxylase (GAD), (2) glutamate transaminase (GAT) converting α-ketoglutarate (α-KG) to GABA, and (3) by conversion of putrescine to GABA via aminobutyraldehyde dehydrogenase (ABD) (Figure 9). Rb cells utilize either all of these biosynthetic pathways or use one of them selectively for the GABA synthesis.^24^ Studies have depicted an aberrant expression of GAD in different cancers which reorients the glutamine metabolism toward the GABA synthesis and thereby results in secretion of GABA.^76,77^ Secreted forms of GABA play vital roles in (1) activating GABA receptors present on tumor cells, thereby facilitating the inactivation of GSK-3β which in turn results in increased β-catenin signaling and tumor growth, and (2) blocking the infiltration of CD8+ T-cells in tumor.^76^ It is noteworthy that β-catenin activation is positively correlated to Rb progression and survival. Reports have elucidated the role of the GABA/GABA receptor system in escaping immunosurveillance by tumor cells in differential solid tumors.^77^ Tian et al. have speculated the potency of secreting GABA by neuroblastoma and glioblastoma, which contributes toward the tumor cell proliferation and developing immunosuppressive environment for the progression of these tumors.^78−82^ Henceforth, the close association between the increased GABA levels in retinoblastoma with its development of immune resistance and tumor growth progression can be anticipated.

### 3.2 Specificity of Metabolic Patterns of Bilateral Rb Patients

Compared to controls and unilateral Rb cases, bilateral Rb patients showed significantly increased serum levels of creatine, biotin, methionine, arginine, putrescine, and myoinositol. High levels of creatine have been associated with accelerated cancer progression.^83,84^ Creatine serves as a methyl donor, which is suggested to participate in methylation of oncoprotein PPA2 which in turn is linked with the activation of pathways related to cell proliferation and neoplastic transformation.^85,86^ Changes in serum creatine levels in Rb patients may also be correlated to amended ATP homeostasis.^87^ High energy requirements correlate with high creatine requirements in Rb cells. Hence, to meet high creatine levels in bilateral Rb, higher production of creatine by the liver and its continuous release in blood are expected to maintain the homeostasis. Biotin is a carrier of activated CO_2_ and serves as an important cofactor for the enzymes involved in carboxylation reactions.^88^ The reason for high levels of biotin in the serum of bilateral Rb patients is unknown, but a simple explanation may be that Rb cells have lost their ability to absorb this vitamin. This is in line with a previous study that observed altered biotin levels in adenocarcinoma cells in patients suffering from colorectal adenocarcinoma.^89^

Consistent with other cancers including colorectal cancer and ovarian cancer, high levels of methionine in Rb patients have also been detected.^90,91^ Cancer cells are dependent on exogenous methionine for methylation reaction.^92^ Limited exogenous methionine supply has also been associated with decreased cancer cell proliferation.^92^ Both *in vivo* and *in vitro* studies implied that the restricted exogenous methionine acts as an inhibitor for cancer cell growth and thus can serve as a potential approach in cancer treatment.^93,94^ Congruent with the above reports, this study also revealed increased methionine levels in Rb patients. Ascorbic acid is a micronutrient that plays an imperative role as an antioxidant at physiological concentrations and also participates in cancer metabolic activities.^95^ Higher levels of ascorbic acid were found in serum from Rb patients, indicating the decreased uptake of vitamin C by Rb cells or due to its substantial extrusion in the extracellular environment by the retinal cells owing to the deteriorated metabolic activities associated with Rb progression, as normal retina encompasses 100-fold more concentration of ascorbate than in blood serum.^96^ Indeed, high doses of ascorbic acid along with chemotherapy have adverse effects on Rb cells implying that Rb tumors are reluctant to uptake ascorbic acid, for their own survivability.^97−100^

Arginine metabolism plays an essential role in cancer progression and tumor biology.^101^ Normal cells are not completely dependent on external arginine; however, many cancer cells are addicted to external arginine. It has been marked that ASS1 (arginosuccinate synthase 1) gene encoding for arginosuccinate synthase 1, is silenced or nonfunctional in some human cancers including Rb.^102^ This indicates that the Rb cells are also arginine auxotrophs that depend on external circulating arginine to cope with its increasing arginine requirements. Further, the enzyme arginine deiminase (ADI), which catalyzes arginine into citrulline and ammonia, has been shown to inhibit the growth of Rb cells and induce the Rb cell death and apoptosis both in the presence and absence of arginine containing media. This implies that ADI is metabolizing the arginine available for the rapidly growing Rb cells that resulted in arginine depletion.^102^ Additionally, it has been proposed that the arginine deiminase (ADI) enzyme can suppress the oncogenic activity of Rb cells by depriving the arginine required for their growth. Hence, it has been postulated that the depletion of arginine by ADI treatment can serve as a potential treatment strategy to combat Rb.^102^ Indeed, ADI-based therapy has also gained tremendous attention to treat a variety of other cancers.^103−106^ Altogether, increased arginine levels in serum of Rb patients indicate that circulating arginine is required to cope with the continuously increasing demand of Rb cells for their growth and proliferation owing to their lack of ASS1 activity and overexpression of ADI enzyme. These findings are in line with Sahoo et al., who have also marked preferential utilization of arginine by Rb tumors.^24^ Putrescine is one of the important polyamines present in humans, produced via arginine metabolism. Putrescine levels have been correlated with cell proliferation, and also in direct correlation with the size of malignant tumors associated with the central nervous system, including brain tumors.^107,108^ Increased serum putrescine levels have also been elucidated in several other cancers including prostate and lung cancers.^109^ Ornithine decarboxylase (ODC) is involved in transforming ornithine into putrescine.^110^ Owing to the upregulation of ODC in different cancer cell types, attempts were made by Muthukumaran et al. to silence the ornithine decarboxylase enzyme in Rb cells, which resulted in reduced putrescine levels in Rb cells that conferred modulatory effects on cell proliferation.^111^ This implies altered activities of ODC in retinoblastoma cells as well, which can be associated with increased polyamine synthesis. This could be one of the reasons for high levels of putrescine in Rb patients, in contrast to healthy individuals. Further, increased levels of myoinositol have been marked in Rb patients, which implies the deregulation of inositol metabolic pathways. Myoinositol is an osmolyte that participates in cell-signaling events. Increased expressions of myoinositol were also evident in medulloblastoma and neuroblastoma.^22^ Altogether, these metabolic alterations have a potency to discriminate Rb patients from controls and are also capable of distinguishing unilateral and bilateral Rb cases. Additionally, such metabolic distortions have highlighted some of the biological pathways perturbed in Rb and thereby assisted in understanding the Rb pathogenesis. Some of the distinct metabolic signatures can be utilized for future Rb diagnostic and prognostic purposes, followed by cross-validation studies. Particularly, a variety of contemporary metabolites including 2-phosphoglycerate, 4-aminobutyrate, leucine, proline, creatine, putrescine, and arginine and their associated metabolic pathways have been identified with the potential to block or revert the growth of Rb. Attempts have been made to block the availability of various metabolites to hinder cancer cell growth.^112−114^ On this note, arginine and putrescine availability to Rb cells has been blocked/targeted by employing ADI treatment approach and ODC silencing strategy using cell-based studies.^111,115^ Such metabolite silencing has been proposed as a prospective Rb treatment strategy. Furthermore, the biomarkers attained from our current discovery metabolomics have a potency to execute future reprogramming metabolomics to evaluate the reprogramming efficacy of exogenous metabolite treatment to Rb patients.

## 4 Conclusions

In summary, the present study depicted metabolic reprogramming in Rb patients on the basis of metabolic discrepancies observed in serum samples of Rb patients with respect to controls. Further, differential circulatory metabolites among the unilateral and bilateral Rb patients suggested the high degree of disease severity associated with bilateral Rb cases, as expected. Intense metabolic alterations in bilateral Rb imply the augmented altered energy metabolism, increased oxidative stress, and adaptive amino acid utilization/synthesis to support anabolic/catabolic activities and cancer-induced inflammation. Metabolic biomarkers associated with unilateral/bilateral Rb patients identified in the present study have potential to lay the foundation for future studies focusing on (1) the development of therapeutic interventions targeting Rb and (2) correlating the mechanisms of metabolic perturbations with respect to clinical medication. Sample size and patient heterogeneity to a certain extent limit the overall conclusions of the current study. Hence, in order to translate these findings for the development of serum-based diagnostic markers and clinical management of Rb, and its subtypes, future studies need to be conducted on a large, varied data set of Rb patients, especially in each of the clinical Rb subtype cohorts integrated with other omics approaches.

## 5 Materials and Methods

### 5.1 Sample Collection

This study was conducted after attaining approval from the institutional review board (IRB) (LEC-BHR-P-01-21-575) of the LV Prasad Eye Institute (LVPEI) and was carried out in accordance with the Declaration of Helsinki guidelines. Written conscious consent was procured from all of the parents/guardians of the patients participating in the study. Rb was diagnosed on the basis of clinical findings examined under anesthetic conditions, B-scan ultrasonography, and orbital imaging. Blood samples were collected from 24 Rb patients, of which 20 patients were less than or equal to 3 years of age (categorized as RL3), and 4 were more than 3 years of age (categorized as RG3). Of 20 Rb patients (RL3), 13 had unilateral and 7 had bilateral Rb. For comparative evaluation, blood samples were also collected from 26 patients with no retinal pathology. These samples served as controls, of which 11 patients were less than or equal to 3 (belongs to CL3), and 15 were greater than 3 years of age (belongs to CG3) (Table S1). This study involved the use of serum samples; hence, 2 mL of blood was collected from each patient in serum tubes. All of the blood samples were centrifuged at 2000*g* for 15 min at 4 °C in order to separate the cellular debris. The supernatant (which represents the cell-free serum sample) was transferred to a 1.5 mL microcentrifuge tube and stored at −80 °C. 300 μL of serum samples were lyophilized and preserved at −80 °C until further experiments.

### 5.2 NMR Measurements

Lyophilized serum samples were dissolved in 600 μL of 20 mM salined sodium phosphate buffer, pH 7.4 (prepared in 100% D_2_O, containing 0.9% sodium chloride). NMR samples were centrifuged at 10 000 rpm (6708*g*) at room temperature for 5 min. 500 μL of the sample was added to a 5 mm NMR tube. A coaxial tube consisting of 60 μL of 1 mM TSP (a chemical shift indicator) was inserted into the NMR tube. The final concentration of TSP was 100 μM.^116^ All of the ^1^H NMR spectral measurements on serum samples were acquired at 298 K using a Bruker 500 MHz NMR spectrometer. One-dimensional ^1^H NMR spectra on each serum sample were recorded using cpmgpr1d (Carr–Purcell–Meiboom–Gill (CPMG)) pulse sequence from the standard Bruker library with water presaturation.^117−119^ For each sample, the spectrum was recorded with 1536 acquisition scans, a spectral width of 20 ppm, and a relaxation delay of 4 s. A relaxation delay (d1) of 4 s was used to obtain accurate measurements and overcome the systematic errors resulting from relaxation effects, which can cause attenuation in the intensity profiles.

### 5.3 NMR Data Processing and Metabolite Profiling

Each CPMG-NMR spectrum acquired was manually processed by a standard Fourier transformation protocol in TopSpin 4.0.6, software designed for NMR data processing developed by Bruker. Further, each spectrum was calibrated with respect to the TSP signal (singlet) at 0 ppm, followed by phase and baseline corrections. Identification and assignment of chemical shifts in 1D ^1^H NMR spectra were performed using the Chenomx NMR suite 8.4 (Chenomx Inc., Edmonton, AB, Canada). This software employs a 500 MHz NMR chemical shift database and publicly available human metabolome database (HMDB).^120^

### 5.4 Statistical Analysis

Statistical assessment of alterations in the metabolic profiles of serum samples from Rb patients and controls was performed by exporting the metabolite concentration data set to the web-based MetaboAnalyst (V5.0) server (https://www.metaboanalyst.ca).^121^ Metabolite(s) concentration data set was preprocessed using interquartile range data filtering, sample normalization using median, accompanied with square root transformation, followed by pareto scaling.^121−123^ The normalized data set was analyzed using the univariate and multivariate statistical analyses in order to highlight the metabolic differences between the Rb patients and controls. Univariate analysis was performed using the Log2 fold change and *t* test to identify the statistically significant metabolites exhibiting the relative fold change (FC ≥ 1.4) in their concentrations among the two groups.^124,125^

Multivariate statistical analysis, which includes principal component analysis (PCA), and partial least-squares discriminant analysis (PLS-DA) were conducted to identify the imperative metabolites contributing to the discrimination of the two groups. PCA is an unsupervised statistical analysis method which was used to obtain an overall grouping trend and to decode the outliers on the basis of variance across the data set. Following an initial overview and determination of the outliers, PLS-DA, a supervised clustering method, was employed to identify the significant features contributing to group discrimination. A 10-fold cross-validation algorithm was employed to validate the PLS-DA model to avert data overfitting. Three important parameters including (*R*^2^) sum of squares captured by model, prediction accuracy, and *Q*^2^ (cross-validated *R*^2^) were taken into consideration to assess the attained model. Further, the variable importance on projection (VIP) score (>1) was used to define the discriminatory metabolites between the Rb patients and normal control subjects. Based on the VIP score, the box and whisker plots were generated for the topmost discriminatory metabolites with VIP score >1. Additionally, a popular machine learning algorithm known as random forest (RF) classification contained in the statistical analysis package of the MetaboAnalyst server was also performed. RF also has the potential of ranking the discriminatory metabolites in accordance with their contribution toward the classification accuracy. Classification accuracy is measured in terms of mean decrease accuracy (MDA) that calculates the significance of a discriminatory metabolite on the basis of assessment of variations in the prediction accuracy parameter when the values of the variables are permuted in a random fashion in contrast to original observations.^126^ Metabolites with MDA values ≥0.01 were considered as metabolites with discriminatory potency and were further appraised by employing Student’s *t* test with statistical significance *p*-value threshold set at *p* < 0.05. A heat map showing all of the assigned metabolites along with their differential concentrations in both Rb patients and controls was generated using an unsupervised hierarchical clustering method which relies on Euclidean and Ward linkage algorithms.

To correlate the functionality of discriminatory metabolites obtained using multivariate analysis, pathway impact analysis was carried out by exporting the data to the pathway analysis module contained in MetaboAnalyst.^127^ Pathway impact values and −log10(*p*) values were calculated based on pathway topology and pathway enrichment analysis, respectively.^117,119^ KEGG pathway-based inbuilt metabolic library of *Homo sapiens* was employed to identify connections between the altered pathways. A scatter plot exhibiting the significant pathways was generated using the global test enrichment method and relative between-ness centrality parameters for topological analysis.^128^

## Supporting Information Available

Representative principal component analysis (PCA) (Figure S1); performance measurements for the PLS-DA model generated (Figures S2, S5); heat map of discriminatory metabolites (Figures S3, S6–S8); multivariate analysis (Figures S4, S9–S10); Chenomx profiling (Figure S11); clinical details of Rb patients and controls (Table S1); list of metabolites assigned (Table S2); comparative analysis of significantly altered metabolic pathways (Table S3); comparative analysis of topmost metabolites with VIP score ≥1 (Table S4); pairwise pathway impact analysis (Tables S5–S8) (PDF)

## Notes

The study was conducted according to the guidelines of the Declaration of Helsinki and approved by the Institutional Ethics Committee of L V Prasad Eye Institute (protocol code: LEC-BHR-P-01-21-575, approved: 22 January 2021).

## References

[ref1] Albert D. M. Historic review of retinoblastoma. Ophthalmology 1987, 94 (6), 654–662. 10.1016/S0161-6420(87)33407-4. 3306547

[ref2] Mendoza P. R.; Grossniklaus H. E. The Biology of Retinoblastoma. Prog. Mol. Biol. Transl. Sci. 2015, 134, 503–516. 10.1016/bs.pmbts.2015.06.012. 26310174

[ref3] Satyanarayana L.; Asthana S.; Labani S. P. Childhood cancer incidence in India: a review of population-based cancer registries. Indian Pediatr. 2014, 51 (3), 218–220. 10.1007/s13312-014-0377-0. 24736911

[ref4] Nichols K. E.; Walther S.; Chao E.; Shields C.; Ganguly A. Recent advances in retinoblastoma genetic research. Curr. Opin. Ophthalmol. 2009, 20 (5), 351–355. 10.1097/ICU.0b013e32832f7f25. 19587599

[ref5] Knudson A. G. Jr. Mutation and cancer: statistical study of retinoblastoma. Proc. Natl. Acad. Sci. U.S.A. 1971, 68 (4), 820–823. 10.1073/pnas.68.4.820. 5279523 PMC389051

[ref6] Lohmann D. R.; Gallie B. L. Retinoblastoma. In GeneReviews((R)); Adam M. P.; Mirzaa G. M.; Pagon R. A.; Wallace S. E.; Bean L. J. H.; Gripp K. W.; Amemiya A., Eds.; University of Washington: Seattle (WA), 1993. 20301625

[ref7] Kleinerman R. A.; Yu C. L.; Little M. P.; Li Y.; Abramson D.; Seddon J.; Tucker M. A. Variation of second cancer risk by family history of retinoblastoma among long-term survivors. J. Clin. Oncol. 2012, 30 (9), 950–957. 10.1200/JCO.2011.37.0239. 22355046 PMC3341108

[ref8] Kitajima S.; Takahashi C. Intersection of retinoblastoma tumor suppressor function, stem cells, metabolism, and inflammation. Cancer Sci. 2017, 108 (9), 1726–1731. 10.1111/cas.13312. 28865172 PMC5581511

[ref9] Burkhart D. L.; Sage J. Cellular mechanisms of tumour suppression by the retinoblastoma gene. Nat. Rev. Cancer 2008, 8 (9), 671–682. 10.1038/nrc2399. 18650841 PMC6996492

[ref10] Engel B. E.; Cress W. D.; Santiago-Cardona P. G. The Retinoblastoma Protein: A Master Tumor Suppressor Acts as a Link between Cell Cycle and Cell Adhesion. Cell Health Cytoskelet. 2015, 7, 1–10. 10.2147/chc.s28079. 28090172 PMC5228373

[ref11] Chinnam M.; Goodrich D. W. RB1, development, and cancer. Curr. Top. Dev. Biol. 2011, 94, 129–169. 10.1016/B978-0-12-380916-2.00005-X. 21295686 PMC3691055

[ref12] Dyson N. J. RB1: a prototype tumor suppressor and an enigma. Genes Dev. 2016, 30 (13), 1492–1502. 10.1101/gad.282145.116. 27401552 PMC4949322

[ref13] Knudsen E. S.; Knudsen K. E. Tailoring to RB: tumour suppressor status and therapeutic response. Nat. Rev. Cancer 2008, 8 (9), 714–724. 10.1038/nrc2401. 19143056 PMC2914856

[ref14] Hanahan D.; Weinberg R. A. Hallmarks of cancer: the next generation. Cell 2011, 144 (5), 646–674. 10.1016/j.cell.2011.02.013. 21376230

[ref15] Xia L.; Oyang L.; Lin J.; Tan S.; Han Y.; Wu N.; Yi P.; Tang L.; Pan Q.; Rao S.; Liang J.; Tang Y.; Su M.; Luo X.; Yang Y.; Shi Y.; Wang H.; Zhou Y.; Liao Q. The cancer metabolic reprogramming and immune response. Mol. Cancer 2021, 20 (1), 28 10.1186/s12943-021-01316-8. 33546704 PMC7863491

[ref16] Qiu S.; Cai Y.; Yao H.; Lin C.; Xie Y.; Tang S.; Zhang A. Small molecule metabolites: discovery of biomarkers and therapeutic targets. Signal Transduction Targeted Ther. 2023, 8 (1), 132 10.1038/s41392-023-01399-3. PMC10026263 36941259

[ref17] Schmidt D. R.; Patel R.; Kirsch D. G.; Lewis C. A.; Vander Heiden M. G.; Locasale J. W. Metabolomics in cancer research and emerging applications in clinical oncology. CA-Cancer J. Clin. 2021, 71 (4), 333–358. 10.3322/caac.21670. 33982817 PMC8298088

[ref18] Shi R.; Tang Y. Q.; Miao H. Metabolism in tumor microenvironment: Implications for cancer immunotherapy. MedComm 2020, 1 (1), 47–68. 10.1002/mco2.6. 34766109 PMC8489668

[ref19] Babu V. S.; Mallipatna A.; Sa D.; Dudeja G.; Kannan R.; Shetty R.; Nair A. P.; Gundimeda S.; Chaurasia S. S.; Verma N. K.; Lakshminarayanan R.; Heymans S.; Barathi V. A.; Guha N.; Ghosh A. Integrated Analysis of Cancer Tissue and Vitreous Humor from Retinoblastoma Eyes Reveals Unique Tumor-Specific Metabolic and Cellular Pathways in Advanced and Non-Advanced Tumors. Cells 2022, 11 (10), 1668 10.3390/cells11101668. 35626705 PMC9139581

[ref20] Guha N.; Livi C.; Babu V. S.; Winer L.; SA D.; Lateef S.; Gundimeda S.; Padmanabhan A.; Dranka B.; Ghosh A. Abstract 2515: Understanding metabolic dysfunction in retinoblastoma development. Cancer Res. 2017, 77 (13_Supplement), 2515 10.1158/1538-7445.am2017-2515.

[ref21] Kohe S.; Brundler M. A.; Jenkinson H.; Parulekar M.; Wilson M.; Peet A. C.; McConville C. M.; Children’s C.; Leukaemia G.; Metabolite profiling in retinoblastoma identifies novel clinicopathological subgroups. Br. J. Cancer 2015, 113 (8), 1216–1224. 10.1038/bjc.2015.318. 26348444 PMC4647873

[ref22] Kohe S. E.; Bennett C. D.; Gill S. K.; Wilson M.; McConville C.; Peet A. C. Metabolic profiling of the three neural derived embryonal pediatric tumors retinoblastoma, neuroblastoma and medulloblastoma, identifies distinct metabolic profiles. Oncotarget 2018, 9 (13), 11336–11351. 10.18632/oncotarget.24168. 29541417 PMC5834290

[ref23] Liu W.; Luo Y.; Dai J.; Yang L.; Huang L.; Wang R.; Chen W.; Huang Y.; Sun S.; Cao J.; Wu J.; Han M.; Fan J.; He M.; Qian K.; Fan X.; Jia R. Monitoring Retinoblastoma by Machine Learning of Aqueous Humor Metabolic Fingerprinting. Small Methods 2022, 6 (1), e2101220 10.1002/smtd.202101220. 35041286

[ref24] Sahoo S.; Ravi Kumar R. K.; Nicolay B.; Mohite O.; Sivaraman K.; Khetan V.; Rishi P.; Ganesan S.; Subramanyan K.; Raman K.; Miles W.; Elchuri S. V. Metabolite systems profiling identifies exploitable weaknesses in retinoblastoma. FEBS Lett. 2019, 593 (1), 23–41. 10.1002/1873-3468.13294. 30417337

[ref25] Bar N.; Korem T.; Weissbrod O.; Zeevi D.; Rothschild D.; Leviatan S.; Kosower N.; Lotan-Pompan M.; Weinberger A.; Le Roy C. I.; Menni C.; Visconti A.; Falchi M.; Spector T. D.; consortium I. D.; Adamski J.; Franks P. W.; Pedersen O.; Segal E.; et al. A reference map of potential determinants for the human serum metabolome. Nature 2020, 588 (7836), 135–140. 10.1038/s41586-020-2896-2. 33177712

[ref26] Fan S.; Shahid M.; Jin P.; Asher A.; Kim J. Identification of Metabolic Alterations in Breast Cancer Using Mass Spectrometry-Based Metabolomic Analysis. Metabolites 2020, 10 (4), 170 10.3390/metabo10040170. 32344578 PMC7241246

[ref27] Ikeda A.; Nishiumi S.; Shinohara M.; Yoshie T.; Hatano N.; Okuno T.; Bamba T.; Fukusaki E.; Takenawa T.; Azuma T.; Yoshida M. Serum metabolomics as a novel diagnostic approach for gastrointestinal cancer. Biomed. Chromatogr. 2012, 26 (5), 548–558. 10.1002/bmc.1671. 21773981

[ref28] Shekher A.; Puneet; Awasthee N.; Kumar U.; Raj R.; Kumar D.; Gupta S. C. Association of altered metabolic profiles and long non-coding RNAs expression with disease severity in breast cancer patients: analysis by (1)H NMR spectroscopy and RT-q-PCR. Metabolomics 2023, 19 (2), 2738 10.1007/s11306-023-01972-5. 36710275

[ref29] Singh A.; Prakash V.; Gupta N.; Kumar A.; Kant R.; Kumar D. Serum Metabolic Disturbances in Lung Cancer Investigated through an Elaborative NMR-Based Serum Metabolomics Approach. ACS Omega 2022, 7 (6), 5510–5520. 10.1021/acsomega.1c06941. 35187366 PMC8851899

[ref30] Wang W.; Rong Z.; Wang G.; Hou Y.; Yang F.; Qiu M. Cancer metabolites: promising biomarkers for cancer liquid biopsy. Biomark Res. 2023, 11 (1), 66 10.1186/s40364-023-00507-3. 37391812 PMC10311880

[ref31] Jung J.; Jung Y.; Bang E. J.; Cho S. I.; Jang Y. J.; Kwak J. M.; Ryu D. H.; Park S.; Hwang G. S. Noninvasive diagnosis and evaluation of curative surgery for gastric cancer by using NMR-based metabolomic profiling. Ann. Surg. Oncol. 2014, 21, 736–742. 10.1245/s10434-014-3886-0. 25092158

[ref32] Mirnezami R.; Jimenez B.; Li J. V.; Kinross J. M.; Veselkov K.; Goldin R. D.; Holmes E.; Nicholson J. K.; Darzi A. Rapid diagnosis and staging of colorectal cancer via high-resolution magic angle spinning nuclear magnetic resonance (HR-MAS NMR) spectroscopy of intact tissue biopsies. Ann. Surg. 2014, 259 (6), 1138–1149. 10.1097/SLA.0b013e31829d5c45. 23860197

[ref33] Rocha C. M.; Barros A. S.; Goodfellow B. J.; Carreira I. M.; Gomes A.; Sousa V.; Bernardo J.; Carvalho L.; Gil A. M.; Duarte I. F. NMR metabolomics of human lung tumours reveals distinct metabolic signatures for adenocarcinoma and squamous cell carcinoma. Carcinogenesis 2015, 36 (1), 68–75. 10.1093/carcin/bgu226. 25368033

[ref34] Wang A. S.; Lodi A.; Rivera L. B.; Izquierdo-Garcia J. L.; Firpo M. A.; Mulvihill S. J.; Tempero M. A.; Bergers G.; Ronen S. M. HR-MAS MRS of the pancreas reveals reduced lipid and elevated lactate and taurine associated with early pancreatic cancer. NMR Biomed. 2014, 27 (11), 1361–1370. 10.1002/nbm.3198. 25199993 PMC5554431

[ref35] Wilson M.; Davies N. P.; Brundler M. A.; McConville C.; Grundy R. G.; Peet A. C. High resolution magic angle spinning 1H NMR of childhood brain and nervous system tumours. Mol. Cancer 2009, 8, 6 10.1186/1476-4598-8-6. 19208232 PMC2651110

[ref36] Guleria A.; Kumar A.; Kumar U.; Raj R.; Kumar D. NMR Based Metabolomics: An Exquisite and Facile Method for Evaluating Therapeutic Efficacy and Screening Drug Toxicity. Curr. Top. Med. Chem. 2018, 18 (20), 1827–1849. 10.2174/1568026619666181120141603. 30465509

[ref37] Baldassarre M. E.; Laforgia N. Metabolomics Applications in Children: A Right Way to Go. Metabolites 2020, 10 (9), 364 10.3390/metabo10090364. 32911604 PMC7569896

[ref38] Chiu C. Y.; Yeh K. W.; Lin G.; Chiang M. H.; Yang S. C.; Chao W. J.; Yao T. C.; Tsai M. H.; Hua M. C.; Liao S. L.; Lai S. H.; Cheng M. L.; Huang J. L. Metabolomics Reveals Dynamic Metabolic Changes Associated with Age in Early Childhood. PLoS One 2016, 11 (2), e0149823 10.1371/journal.pone.0149823. 26914934 PMC4767415

[ref39] Freitas-Fernandes L. B.; Fontes G. P.; Letieri A. D. S.; Valente A. P.; Souza I. P. R.; Fidalgo T. NMR-Based Metabolomics Demonstrates a Metabolic Change during Early Developmental Stages from Healthy Infants to Young Children. Metabolites 2023, 13 (3), 445 10.3390/metabo13030445. 36984885 PMC10058828

[ref40] Moco S.; Collino S.; Rezzi S.; Martin F. P. Metabolomics perspectives in pediatric research. Pediatr. Res. 2013, 73 (4 Pt 2), 570–576. 10.1038/pr.2013.1. 23314292

[ref41] van Beijsterveldt I. A.; Snowden S. G.; Myers P. N.; de Fluiter K. S.; van de Heijning B.; Brix S.; Ong K. K.; Dunger D. B.; Hokken-Koelega A. C. S.; Koulman A. Metabolomics in early life and the association with body composition at age 2 years. Pediatr. Obes. 2022, 17 (3), e12859 10.1111/ijpo.12859. 34644810 PMC9286420

[ref42] Stenfelt S.; Blixt M. K. E.; All-Ericsson C.; Hallbook F.; Boije H. Heterogeneity in retinoblastoma: a tale of molecules and models. Clin. Transl. Med. 2017, 6 (1), 42 10.1186/s40169-017-0173-2. 29124525 PMC5680409

[ref43] Suresh Babu V.; Dudeja G.; Sa D.; Bisht A.; Shetty R.; Heymans S.; Guha N.; Ghosh A. Lack of Retinoblastoma Protein Shifts Tumor Metabolism from Glycolysis to OXPHOS and Allows the Use of Alternate Fuels. Cells 2022, 11 (20), 3182 10.3390/cells11203182. 36291051 PMC9600484

[ref44] Faubert B.; Solmonson A.; DeBerardinis R. J. Metabolic reprogramming and cancer progression. Science 2020, 368 (6487), 3930 10.1126/science.aaw5473. PMC7227780 32273439

[ref45] Schiliro C.; Firestein B. L. Mechanisms of Metabolic Reprogramming in Cancer Cells Supporting Enhanced Growth and Proliferation. Cells 2021, 10 (5), 1056 10.3390/cells10051056. 33946927 PMC8146072

[ref46] Saito R. d. F.; Andrade L. N. S.; Bustos S. O.; Chammas R. Phosphatidylcholine-Derived Lipid Mediators: The Crosstalk Between Cancer Cells and Immune Cells. Front. Immunol. 2022, 13, 768606 10.3389/fimmu.2022.768606. 35250970 PMC8889569

[ref47] Viswanath P.; Radoul M.; Izquierdo-Garcia J. L.; Luchman H. A.; Gregory Cairncross J.; Pieper R. O.; Phillips J. J.; Ronen S. M. Mutant IDH1 gliomas downregulate phosphocholine and phosphoethanolamine synthesis in a 2-hydroxyglutarate-dependent manner. Cancer Metab. 2018, 6, 3 10.1186/s40170-018-0178-3. 29619216 PMC5881177

[ref48] Li Z.; Vance D. E. Phosphatidylcholine and choline homeostasis. J. Lipid Res. 2008, 49 (6), 1187–1194. 10.1194/jlr.R700019-JLR200. 18204095

[ref49] de la Cruz-López K. G.; Castro-Munoz L. J.; Reyes-Hernandez D. O.; Garcia-Carranca A.; Manzo-Merino J. Lactate in the Regulation of Tumor Microenvironment and Therapeutic Approaches. Front. Oncol. 2019, 9, 1143 10.3389/fonc.2019.01143. 31737570 PMC6839026

[ref50] Li X.; Yang Y.; Zhang B.; Lin X.; Fu X.; An Y.; Zou Y.; Wang J. X.; Wang Z.; Yu T. Lactate metabolism in human health and disease. Signal Transduction Targeted Ther. 2022, 7 (1), 305 10.1038/s41392-022-01151-3. PMC9434547 36050306

[ref51] Bonuccelli G.; Tsirigos A.; Whitaker-Menezes D.; Pavlides S.; Pestell R. G.; Chiavarina B.; Frank P. G.; Flomenberg N.; Howell A.; Martinez-Outschoorn U. E.; Sotgia F.; Lisanti M. P. Ketones and lactate ″fuel″ tumor growth and metastasis: Evidence that epithelial cancer cells use oxidative mitochondrial metabolism. Cell Cycle 2010, 9 (17), 3506–3514. 10.4161/cc.9.17.12731. 20818174 PMC3047616

[ref52] Walenta S.; Wetterling M.; Lehrke M.; Schwickert G.; Sundfor K.; Rofstad E. K.; Mueller-Klieser W. High lactate levels predict likelihood of metastases, tumor recurrence, and restricted patient survival in human cervical cancers. Cancer Res. 2000, 60 (4), 916–921. 10706105

[ref53] Brizel D. M.; Schroeder T.; Scher R. L.; Walenta S.; Clough R. W.; Dewhirst M. W.; Mueller-Klieser W. Elevated tumor lactate concentrations predict for an increased risk of metastases in head-and-neck cancer. Int. J. Radiat. Oncol. Biol. Phys. 2001, 51 (2), 349–353. 10.1016/S0360-3016(01)01630-3. 11567808

[ref54] Sitter B.; Forsmark A.; Solheim O. Elevated Serum Lactate in Glioma Patients: Associated Factors. Front. Oncol. 2022, 12, 831079 10.3389/fonc.2022.831079. 35664752 PMC9161145

[ref55] Naik A.; Decock J. Lactate Metabolism and Immune Modulation in Breast Cancer: A Focused Review on Triple Negative Breast Tumors. Front. Oncol. 2020, 10, 598626 10.3389/fonc.2020.598626. 33324565 PMC7725706

[ref56] Whitaker-Menezes D.; Martinez-Outschoorn U. E.; Lin Z.; Ertel A.; Flomenberg N.; Witkiewicz A. K.; Birbe R. C.; Howell A.; Pavlides S.; Gandara R.; Pestell R. G.; Sotgia F.; Philp N. J.; Lisanti M. P. Evidence for a stromal-epithelial ″lactate shuttle″ in human tumors: MCT4 is a marker of oxidative stress in cancer-associated fibroblasts. Cell Cycle 2011, 10 (11), 1772–1783. 10.4161/cc.10.11.15659. 21558814 PMC3142461

[ref57] Martinez-Outschoorn U. E.; Pavlides S.; Howell A.; Pestell R. G.; Tanowitz H. B.; Sotgia F.; Lisanti M. P. Stromal-epithelial metabolic coupling in cancer: integrating autophagy and metabolism in the tumor microenvironment. Int. J. Biochem. Cell Biol. 2011, 43 (7), 1045–1051. 10.1016/j.biocel.2011.01.023. 21300172 PMC3102770

[ref58] Feron O. Pyruvate into lactate and back: from the Warburg effect to symbiotic energy fuel exchange in cancer cells. Radiother. Oncol. 2009, 92 (3), 329–333. 10.1016/j.radonc.2009.06.025. 19604589

[ref59] Sonveaux P.; Vegran F.; Schroeder T.; Wergin M. C.; Verrax J.; Rabbani Z. N.; De Saedeleer C. J.; Kennedy K. M.; Diepart C.; Jordan B. F.; Kelley M. J.; Gallez B.; Wahl M. L.; Feron O.; Dewhirst M. W. Targeting lactate-fueled respiration selectively kills hypoxic tumor cells in mice. J. Clin Invest 2008, 118 (12), 3930–3942. 10.1172/JCI36843. 19033663 PMC2582933

[ref60] Wu Y.; Dong Y.; Atefi M.; Liu Y.; Elshimali Y.; Vadgama J. V. Lactate, a Neglected Factor for Diabetes and Cancer Interaction. Mediators Inflammation 2016, 2016, 6456018 10.1155/2016/6456018. PMC5203906 28077918

[ref61] Winkler B. S. Glycolytic and oxidative metabolism in relation to retinal function. J. Gen. Physiol. 1981, 77 (6), 667–692. 10.1085/jgp.77.6.667. 6267165 PMC2215447

[ref62] Sharpe M. A.; Ijare O. B.; Baskin D. S.; Baskin A. M.; Baskin B. N.; Pichumani K. The Leloir Cycle in Glioblastoma: Galactose Scavenging and Metabolic Remodeling. Cancers 2021, 13 (8), 1815 10.3390/cancers13081815. 33920278 PMC8069026

[ref63] Nwosu Z. C.; Ward M. H.; Sajjakulnukit P.; Poudel P.; Ragulan C.; Kasperek S.; Radyk M.; Sutton D.; Menjivar R. E.; Andren A.; Apiz-Saab J. J.; Tolstyka Z.; Brown K.; Lee H. J.; Dzierozynski L. N.; He X.; Ps H.; Ugras J.; Nyamundanda G.; Zhang L.; Halbrook C. J.; Carpenter E. S.; Shi J.; Shriver L. P.; Patti G. J.; Muir A.; Pasca di Magliano M.; Sadanandam A.; Lyssiotis C. A. Uridine-derived ribose fuels glucose-restricted pancreatic cancer. Nature 2023, 618 (7963), 151–158. 10.1038/s41586-023-06073-w. 37198494 PMC10232363

[ref64] Peng H.; Wang Y.; Luo W. Multifaceted role of branched-chain amino acid metabolism in cancer. Oncogene 2020, 39 (44), 6747–6756. 10.1038/s41388-020-01480-z. 32978521 PMC7606751

[ref65] Sivanand S.; Vander Heiden M. G. Emerging Roles for Branched-Chain Amino Acid Metabolism in Cancer. Cancer Cell 2020, 37 (2), 147–156. 10.1016/j.ccell.2019.12.011. 32049045 PMC7082774

[ref66] Vettore L.; Westbrook R. L.; Tennant D. A. New aspects of amino acid metabolism in cancer. Br. J. Cancer 2020, 122 (2), 150–156. 10.1038/s41416-019-0620-5. 31819187 PMC7052246

[ref67] Jung M. K.; Okekunle A. P.; Lee J. E.; Sung M. K.; Lim Y. J. Role of Branched-chain Amino Acid Metabolism in Tumor Development and Progression. J. Cancer Prev. 2021, 26 (4), 237–243. 10.15430/JCP.2021.26.4.237. 35047449 PMC8749315

[ref68] Xiong J. Fatty Acid Oxidation in Cell Fate Determination. Trends Biochem. Sci. 2018, 43 (11), 854–857. 10.1016/j.tibs.2018.04.006. 29735398 PMC6204300

[ref69] D’Aniello C.; Patriarca E. J.; Phang J. M.; Minchiotti G. Proline Metabolism in Tumor Growth and Metastatic Progression. Front. Oncol. 2020, 10, 776 10.3389/fonc.2020.00776. 32500033 PMC7243120

[ref70] Phang J. M.; Liu W. Proline metabolism and cancer. Front. Biosci. 2012, 17 (5), 1835–1845. 10.2741/4022. PMC7467630 22201839

[ref71] Phang J. M.; Liu W.; Hancock C. N.; Fischer J. W. Proline metabolism and cancer: emerging links to glutamine and collagen. Curr. Opin. Clin. Nutr. Metab. Care 2015, 18 (1), 71–77. 10.1097/MCO.0000000000000121. 25474014 PMC4255759

[ref72] Amelio I.; Cutruzzola F.; Antonov A.; Agostini M.; Melino G. Serine and glycine metabolism in cancer. Trends Biochem. Sci. 2014, 39 (4), 191–198. 10.1016/j.tibs.2014.02.004. 24657017 PMC3989988

[ref73] Bui B. V.; Hu R. G.; Acosta M. L.; Donaldson P.; Vingrys A. J.; Kalloniatis M. Glutamate metabolic pathways and retinal function. J. Neurochem. 2009, 111 (2), 589–599. 10.1111/j.1471-4159.2009.06354.x. 19702659

[ref74] Lake N. Taurine and GABA in the rat retina during postnatal development. Visual Neurosci. 1994, 11 (2), 253–260. 10.1017/S0952523800001619. 8003452

[ref75] Young S. Z.; Bordey A. GABA’s control of stem and cancer cell proliferation in adult neural and peripheral niches. Physiology 2009, 24, 171–185. 10.1152/physiol.00002.2009. 19509127 PMC2931807

[ref76] Tian J.; Kaufman D. L. The GABA and GABA-Receptor System in Inflammation, Anti-Tumor Immune Responses, and COVID-19. Biomedicines 2023, 11 (2), 254 10.3390/biomedicines11020254. 36830790 PMC9953446

[ref77] Huang D.; Wang Y.; Thompson J. W.; Yin T.; Alexander P. B.; Qin D.; Mudgal P.; Wu H.; Liang Y.; Tan L.; Pan C.; Yuan L.; Wan Y.; Li Q. J.; Wang X. F. Cancer-cell-derived GABA promotes beta-catenin-mediated tumour growth and immunosuppression. Nat. Cell Biol. 2022, 24 (2), 230–241. 10.1038/s41556-021-00820-9. 35145222 PMC8852304

[ref78] Jow F.; Chiu D.; Lim H. K.; Novak T.; Lin S. Production of GABA by cultured hippocampal glial cells. Neurochem. Int. 2004, 45 (2–3), 273–283. 10.1016/j.neuint.2003.11.021. 15145543

[ref79] Kozlov A. S.; Angulo M. C.; Audinat E.; Charpak S. Target cell-specific modulation of neuronal activity by astrocytes. Proc. Natl. Acad. Sci. U.S.A. 2006, 103 (26), 10058–10063. 10.1073/pnas.0603741103. 16782808 PMC1502505

[ref80] Le Meur K.; Mendizabal-Zubiaga J.; Grandes P.; Audinat E. GABA release by hippocampal astrocytes. Front. Comput. Neurosci. 2012, 6, 59 10.3389/fncom.2012.00059. 22912614 PMC3421239

[ref81] Lee M.; Schwab C.; McGeer P. L. Astrocytes are GABAergic cells that modulate microglial activity. Glia 2011, 59 (1), 152–165. 10.1002/glia.21087. 21046567

[ref82] Liu Q. Y.; Schaffner A. E.; Chang Y. H.; Maric D.; Barker J. L. Persistent activation of GABA(A) receptor/Cl(−) channels by astrocyte-derived GABA in cultured embryonic rat hippocampal neurons. J. Neurophysiol. 2000, 84 (3), 1392–1403. 10.1152/jn.2000.84.3.1392. 10980012

[ref83] Ladep N. G.; Dona A. C.; Lewis M. R.; Crossey M. M.; Lemoine M.; Okeke E.; Shimakawa Y.; Duguru M.; Njai H. F.; Fye H. K.; Taal M.; Chetwood J.; Kasstan B.; Khan S. A.; Garside D. A.; Wijeyesekera A.; Thillainayagam A. V.; Banwat E.; Thursz M. R.; Nicholson J. K.; Njie R.; Holmes E.; Taylor-Robinson S. D. Discovery and validation of urinary metabotypes for the diagnosis of hepatocellular carcinoma in West Africans. Hepatology 2014, 60 (4), 1291–1301. 10.1002/hep.27264. 24923488

[ref84] Zhang L.; Bu P. The two sides of creatine in cancer. Trends Cell Biol. 2022, 32 (5), 380–390. 10.1016/j.tcb.2021.11.004. 34895811

[ref85] Guénin S.; Schwartz L.; Morvan D.; Steyaert J. M.; Poignet A.; Madelmont J. C.; Demidem A. PP2A activity is controlled by methylation and regulates oncoprotein expression in melanoma cells: a mechanism which participates in growth inhibition induced by chloroethylnitrosourea treatment. Int. J. Oncol. 2008, 32 (1), 49–57. 10.3892/ijo.32.1.49. 18097542

[ref86] Lécuyer L.; Victor Bala A.; Deschasaux M.; Bouchemal N.; Nawfal Triba M.; Vasson M. P.; Rossary A.; Demidem A.; Galan P.; Hercberg S.; Partula V.; Le Moyec L.; Srour B.; Fiolet T.; Latino-Martel P.; Kesse-Guyot E.; Savarin P.; Touvier M. NMR metabolomic signatures reveal predictive plasma metabolites associated with long-term risk of developing breast cancer. Int. J. Epidemiol. 2018, 47 (2), 484–494. 10.1093/ije/dyx271. 29365091

[ref87] Acosta M. L.; Kalloniatis M.; Christie D. L. Creatine transporter localization in developing and adult retina: importance of creatine to retinal function. Am. J. Physiol. Cell Physiol. 2005, 289 (4), C1015–C1023. 10.1152/ajpcell.00137.2005. 15930147

[ref88] León-Del-Río A. Biotin in metabolism, gene expression, and human disease. J. Inherit. Metab. Dis. 2019, 42 (4), 647–654. 10.1002/jimd.12073. 30746739

[ref89] Cherbonnel-Lasserre C. L.; Linares-Cruz G.; Rigaut J. P.; Sabatier L.; Dutrillaux B. Strong decrease in biotin content may correlate with metabolic alterations in colorectal adenocarcinoma. Int. J. Cancer 1997, 72 (5), 768–775. 10.1002/(SICI)1097-0215(19970904)72:5<768::AID-IJC11>3.0.CO;2-5. 9311592

[ref90] Amir Hashim N. A.; Ab-Rahim S.; Wan Ngah W. Z.; Nathan S.; Ab Mutalib N. S.; Sagap I.; AR A. J.; Mazlan M. Global metabolomics profiling of colorectal cancer in Malaysian patients. Bioimpacts 2021, 11 (1), 33–43. 10.34172/bi.2021.05. 33469506 PMC7803921

[ref91] Wang X.; Zhao X.; Zhao J.; Yang T.; Zhang F.; Liu L. Serum metabolite signatures of epithelial ovarian cancer based on targeted metabolomics. Clin. Chim. Acta 2021, 518, 59–69. 10.1016/j.cca.2021.03.012. 33746017

[ref92] Kaiser P. Methionine Dependence of Cancer. Biomolecules 2020, 10 (4), 568 10.3390/biom10040568. 32276408 PMC7226524

[ref93] Sinha R.; Cooper T. K.; Rogers C. J.; Sinha I.; Turbitt W. J.; Calcagnotto A.; Perrone C. E.; Richie J. P. Jr. Dietary methionine restriction inhibits prostatic intraepithelial neoplasia in TRAMP mice. Prostate 2014, 74 (16), 1663–1673. 10.1002/pros.22884. 25250521

[ref94] Strekalova E.; Malin D.; Good D. M.; Cryns V. L. Methionine Deprivation Induces a Targetable Vulnerability in Triple-Negative Breast Cancer Cells by Enhancing TRAIL Receptor-2 Expression. Clin. Cancer Res. 2015, 21 (12), 2780–2791. 10.1158/1078-0432.CCR-14-2792. 25724522 PMC4470820

[ref95] Chen Z.; Huang Y.; Cao D.; Qiu S.; Chen B.; Li J.; Bao Y.; Wei Q.; Han P.; Liu L. Vitamin C Intake and Cancers: An Umbrella Review. Front. Nutr. 2021, 8, 812394 10.3389/fnut.2021.812394. 35127793 PMC8812486

[ref96] Rose R. C.; Bode A. M. Ocular ascorbate transport and metabolism. Comp. Biochem. Physiol. A Comp. Physiol. 1991, 100 (2), 273–285. 10.1016/0300-9629(91)90470-W. 1685949

[ref97] Blaszczak W.; Barczak W.; Masternak J.; Kopczynski P.; Zhitkovich A.; Rubis B. Vitamin C as a Modulator of the Response to Cancer Therapy. Molecules 2019, 24 (3), 453 10.3390/molecules24030453. 30695991 PMC6384696

[ref98] Mastrangelo D.; Massai L.; Micheli L.; Muscettola M.; Cevenini G. High Doses of Ascorbate Kill Y79 Retinoblastoma Cells In vitro. J. Clin. Exp. Ophthalmol. 2013, 4, 268 10.4172/2155-9570.1000268.

[ref99] Oronowicz J.; Reinhard J.; Reinach P. S.; Ludwiczak S.; Luo H.; Omar Ba Salem M. H.; Kraemer M. M.; Biebermann H.; Kakkassery V.; Mergler S. Ascorbate-induced oxidative stress mediates TRP channel activation and cytotoxicity in human etoposide-sensitive and -resistant retinoblastoma cells. Lab. Invest. 2021, 101 (1), 70–88. 10.1038/s41374-020-00485-2. 32948812 PMC7758186

[ref100] Tomcikova D.; Gerinec A.; Busanyova B.; Husakova K.; Kuniak M.; Autrata R. Incomprehensible treatment of retinoblastoma with high doses of vitamin C. Bratisl Lek Listy 2018, 119 (8), 513–515. 10.4149/BLL_2018_094. 30160161

[ref101] Patil M. D.; Bhaumik J.; Babykutty S.; Banerjee U. C.; Fukumura D. Arginine dependence of tumor cells: targeting a chink in cancer’s armor. Oncogene 2016, 35 (38), 4957–4972. 10.1038/onc.2016.37. 27109103 PMC5457742

[ref102] Kim J. H.; Kim J. H.; Yu Y. S.; Kim D. H.; Min B. H.; Kim K. W. Anti-tumor activity of arginine deiminase via arginine deprivation in retinoblastoma. Oncol. Rep. 2007, 18 (6), 1373–1377. 10.3892/or.18.6.1373. 17982619

[ref103] Fultang L.; Vardon A.; De Santo C.; Mussai F. Molecular basis and current strategies of therapeutic arginine depletion for cancer. Int. J. Cancer 2016, 139 (3), 501–509. 10.1002/ijc.30051. 26913960

[ref104] Delage B.; Fennell D. A.; Nicholson L.; McNeish I.; Lemoine N. R.; Crook T.; Szlosarek P. W. Arginine deprivation and argininosuccinate synthetase expression in the treatment of cancer. Int. J. Cancer 2010, 126 (12), 2762–2772. 10.1002/ijc.25202. 20104527

[ref105] Rogers L. C.; Van Tine B. A. Innate and adaptive resistance mechanisms to arginine deprivation therapies in sarcoma and other cancers. Cancer Drug Resist. 2019, 2 (3), 516–526. 10.20517/cdr.2019.49. 35582579 PMC8992531

[ref106] Riess C.; Shokraie F.; Classen C. F.; Kreikemeyer B.; Fiedler T.; Junghanss C.; Maletzki C. Arginine-Depleting Enzymes - An Increasingly Recognized Treatment Strategy for Therapy-Refractory Malignancies. Cell Physiol. Biochem. 2018, 51 (2), 854–870. 10.1159/000495382. 30466103

[ref107] Harik S. I.; Sutton C. H. Putrescine as a biochemical marker of malignant brain tumors. Cancer Res. 1979, 39 (12), 5010–5015. 227593

[ref108] Lee Y. R.; An K. Y.; Jeon J.; Kim N. K.; Lee J. W.; Hong J.; Chung B. C. Untargeted Metabolomics and Polyamine Profiling in Serum before and after Surgery in Colorectal Cancer Patients. Metabolites 2020, 10 (12), 487 10.3390/metabo10120487. 33260822 PMC7760053

[ref109] Mahmud I.; Hossain S.; Shekhar H. U.; Rahman M. S.; Shahjalal H. M. Relevance of serum polyamine levels in the diagnosis of human prostate and lung cancers. Bangladesh J. Life Sci. 2005, 17 (2), 7–14.

[ref110] Schipper R. G.; Verhofstad A. A. Distribution patterns of ornithine decarboxylase in cells and tissues: facts, problems, and postulates. J. Histochem. Cytochem. 2002, 50 (9), 1143–1160. 10.1177/002215540205000901. 12185192

[ref111] Muthukumaran S.; Bhuvanasundar R.; Umashankar V.; Sulochana K. N. Insights on ornithine decarboxylase silencing as a potential strategy for targeting retinoblastoma. Biomed. Pharmacother. 2018, 98, 23–28. 10.1016/j.biopha.2017.12.030. 29241071

[ref112] Butler M.; van der Meer L. T.; van Leeuwen F. N. Amino Acid Depletion Therapies: Starving Cancer Cells to Death. Trends Endocrinol. Metab. 2021, 32 (6), 367–381. 10.1016/j.tem.2021.03.003. 33795176

[ref113] Sedillo J. C.; Cryns V. L. Targeting the methionine addiction of cancer. Am. J. Cancer Res. 2022, 12 (5), 2249–2276. 35693095 PMC9185618

[ref114] Park I. S.; Kang S. W.; Shin Y. J.; Chae K. Y.; Park M. O.; Kim M. Y.; Wheatley D. N.; Min B. H. Arginine deiminase: a potential inhibitor of angiogenesis and tumour growth. Br. J. Cancer 2003, 89 (5), 907–914. 10.1038/sj.bjc.6601181. 12942125 PMC2394481

[ref115] Kim S.; Song Y. K.; Cho C. S.; Kim H. J.; Fang S.; Jo D. H.; Kim H. Inhibition of protein arginine deiminase II suppresses retinoblastoma in orthotopic transplantation in mice. Oncol. Rep. 2023, 50 (1), 15021 10.3892/or.2023.8583. PMC10285604 37326108

[ref116] Gulati K.; Sarkar S.; Poluri K. M. Metabolomics analysis of complex biological specimens using nuclear magnetic resonance spectroscopy. In Neuromethods; Springer, 2021; Vol. 159, pp 155–171 10.1007/978-1-0716-0864-7_13.

[ref117] Crook A. A.; Powers R. Quantitative NMR-Based Biomedical Metabolomics: Current Status and Applications. Molecules 2020, 25 (21), 5128 10.3390/molecules25215128. 33158172 PMC7662776

[ref118] Nagana Gowda G. A.; Raftery D. NMR-Based Metabolomics. Adv. Exp. Med. Biol. 2021, 1280, 19–37. 10.1007/978-3-030-51652-9_2. 33791972 PMC8816450

[ref119] Wishart D. S. Quantitative metabolomics using NMR. TrAC, Trends Anal. Chem. 2008, 27 (3), 228–237. 10.1016/j.trac.2007.12.001.

[ref120] Wishart D. S.; Guo A.; Oler E.; Wang F.; Anjum A.; Peters H.; Dizon R.; Sayeeda Z.; Tian S.; Lee B. L.; Berjanskii M.; Mah R.; Yamamoto M.; Jovel J.; Torres-Calzada C.; Hiebert-Giesbrecht M.; Lui V. W.; Varshavi D.; Varshavi D.; Allen D.; Arndt D.; Khetarpal N.; Sivakumaran A.; Harford K.; Sanford S.; Yee K.; Cao X.; Budinski Z.; Liigand J.; Zhang L.; Zheng J.; Mandal R.; Karu N.; Dambrova M.; Schioth H. B.; Greiner R.; Gautam V. HMDB 5.0: the Human Metabolome Database for 2022. Nucleic Acids Res. 2022, 50 (D1), D622–D631. 10.1093/nar/gkab1062. 34986597 PMC8728138

[ref121] Pang Z.; Chong J.; Zhou G.; de Lima Morais D. A.; Chang L.; Barrette M.; Gauthier C.; Jacques P. E.; Li S.; Xia J. MetaboAnalyst 5.0: narrowing the gap between raw spectra and functional insights. Nucleic Acids Res. 2021, 49 (W1), W388–W396. 10.1093/nar/gkab382. 34019663 PMC8265181

[ref122] Craig A.; Cloarec O.; Holmes E.; Nicholson J. K.; Lindon J. C. Scaling and normalization effects in NMR spectroscopic metabonomic data sets. Anal. Chem. 2006, 78 (7), 2262–2267. 10.1021/ac0519312. 16579606

[ref123] van den Berg R. A.; Hoefsloot H. C.; Westerhuis J. A.; Smilde A. K.; van der Werf M. J. Centering, scaling, and transformations: improving the biological information content of metabolomics data. BMC Genomics 2006, 7, 142 10.1186/1471-2164-7-142. 16762068 PMC1534033

[ref124] Kairamkonda M.; Sharma M.; Gupta P.; Poluri K. M. Overexpression of bacteriophage T4 and T7 endolysins differentially regulate the metabolic fingerprint of host *Escherichia coli*. Int. J. Biol. Macromol. 2022, 221, 212–223. 10.1016/j.ijbiomac.2022.09.012. 36075302

[ref125] Tripathi S.; Kairamkonda M.; Gupta P.; Poluri K. M. Dissecting the molecular mechanisms of producing biofuel and value-added products by cadmium tolerant microalgae as sustainable biorefinery approach. Chem. Eng. J. 2023, 454, 140068 10.1016/j.cej.2022.140068.

[ref126] Xia J.; Psychogios N.; Young N.; Wishart D. S. MetaboAnalyst: a web server for metabolomic data analysis and interpretation. Nucleic Acids Res. 2009, 37 (Web Server issue), W652–W660. 10.1093/nar/gkp356. 19429898 PMC2703878

[ref127] Chong J.; Wishart D. S.; Xia J. Using MetaboAnalyst 4.0 for Comprehensive and Integrative Metabolomics Data Analysis. Curr. Protoc. Bioinformatics 2019, 68 (1), e86 10.1002/cpbi.86. 31756036

[ref128] Xia J.; Wishart D. S. MSEA: a web-based tool to identify biologically meaningful patterns in quantitative metabolomic data. Nucleic Acids Res. 2010, 38 (Web Server issue), W71–W77. 10.1093/nar/gkq329. 20457745 PMC2896187

