# Supplementary material for: Serum Metabolomics of Retinoblastoma: Assessing the Differential Serum Metabolic Signatures of Unilateral and Bilateral Patients
Source: ACS Omega. 2023 Dec 7;8(50):48233–50. doi: 10.1021/acsomega.3c07424 (PMC10733957; doi:10.1021/acsomega.3c07424)
Supplement: Supplementary file 1 — ao3c07424_si_001.pdf [file ao3c07424_si_001.pdf]

SUPPORTING INFORMATION

Serum metabolomics of retinoblastoma: Assessing the differential serum metabolic signatures of unilateral and bilateral patients

Khushboo Gulati[1,2], Radhika Manukonda[1,2], Manikyaprabhu Kairamkonda[3], Swathi Kaliki[1*], Krishna Mohan Poluri[3,4]*

[1]The Operation Eyesight Universal Institute for Eye Cancer, LV Prasad Eye Institute, Hyderabad-500034, Telangana, India.
[2]Brien Holden Eye Research Center, L. V. Prasad Eye Institute, Hyderabad-500034, Telangana, India.
[3]Department of Biosciences and Bioengineering, Indian Institute of Technology Roorkee, Roorkee-247667, Uttarakhand, India

[4]Centre for Nanotechnology, Indian Institute of Technology Roorkee, Roorkee-247667, Uttarakhand, India

***Corresponding Authors:**
Dr. Swathi Kaliki
The Operation Eyesight Universal Institute for Eye Cancer,
LV Prasad Eye Institute
Hyderabad-500034, Telangana, India.
Email: ***swathikaliki@lvpei.org***

Prof. Krishna Mohan Poluri
Department of Biosciences and Bioengineering
Indian Institute of Technology Roorkee (IIT-Roorkee)
Roorkee-247667, Uttarakhand, INDIA
Email: ***krishna.poluri@bt.iitr.ac.in; mohanpmk@gmail.com***

Figure S1: Multivariate statistical analysis of human serum metabolites from Rb patients and controls: Representative PCA (principal component analysis) score plot depicting the discrimination between the two groups: Controls (pink) and Rb patients' groups (blue).

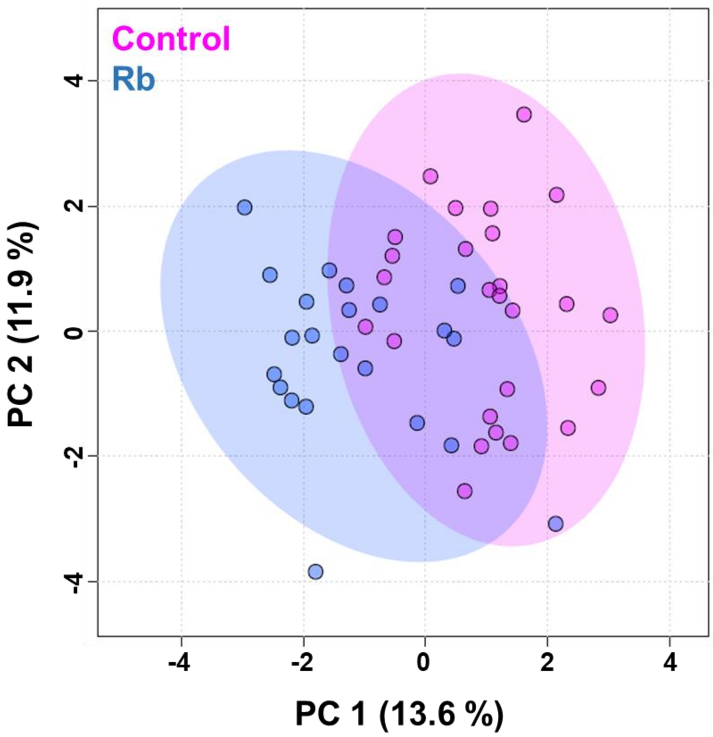

Figure S2: Performance measurements for the PLS-DA model generated for the concentration dataset of 75 metabolites from the serum of controls versus Rb patients: (A) Bar plots presenting the performance parameters (accuracy, goodness of fit (R^2) and predictability (Q^2)) up to the 5th component attained using the 10-fold cross validation. The best classifier of the model is marked as the red star symbol. (B) Cross validation performance measurement values are displayed for all the five components of the PLS-DA model. Validation parameters obtained for the best PLS-DA model include the accuracy of 0.93, R^2 and Q^2 values equal to 0.98 and 0.73 respectively.

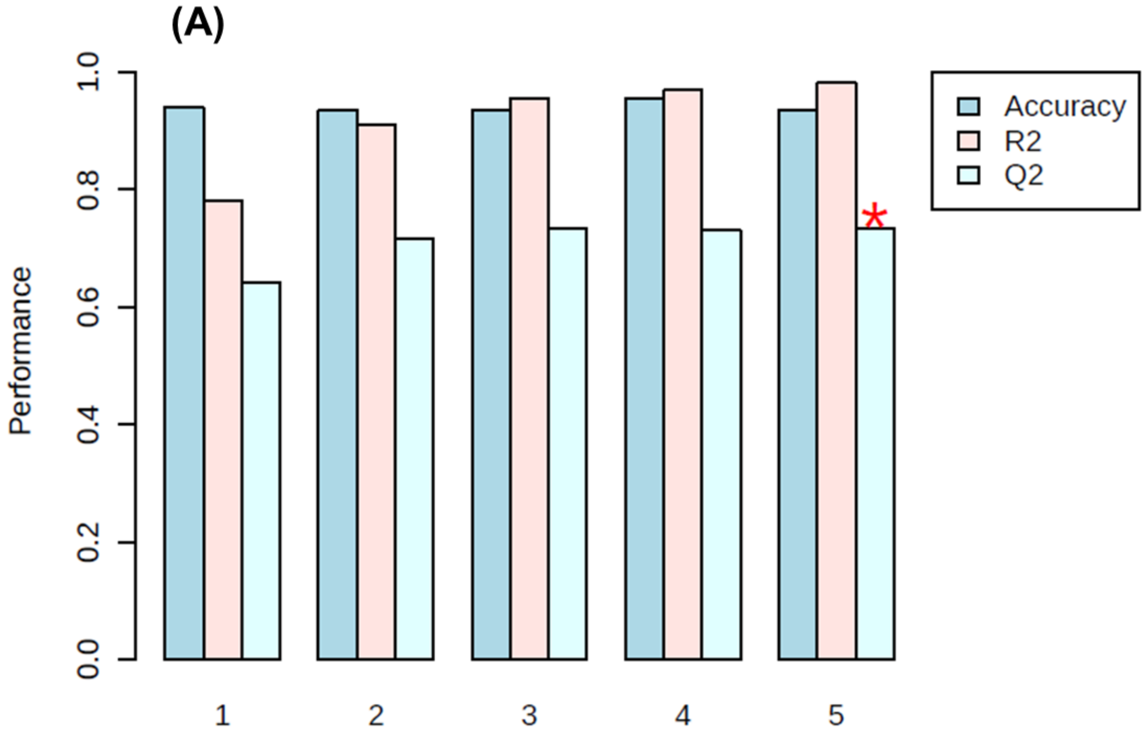

(B)

PLS-DA cross validation details:

Measure	1 comps	2 comps	3 comps	4 comps	5 comps
Accuracy	0.94	0.935	0.935	0.955	0.935
R2	0.78177	0.90951	0.95522	0.96901	0.98192
Q2	0.64149	0.71596	0.7339	0.73091	0.73477

Figure S3: Heat map displaying the z-scores of top 25 discriminatory metabolites among the Rb patients with respect to controls. X-axis represents the serum samples from controls (Ctrl, lanes:1-26) and Rb patients (Rb, lanes: 27- 48). The color gradients signify the differential metabolite concentration in the serum samples, with dark red and dark blue implies the highest and lowest metabolite concentrations.

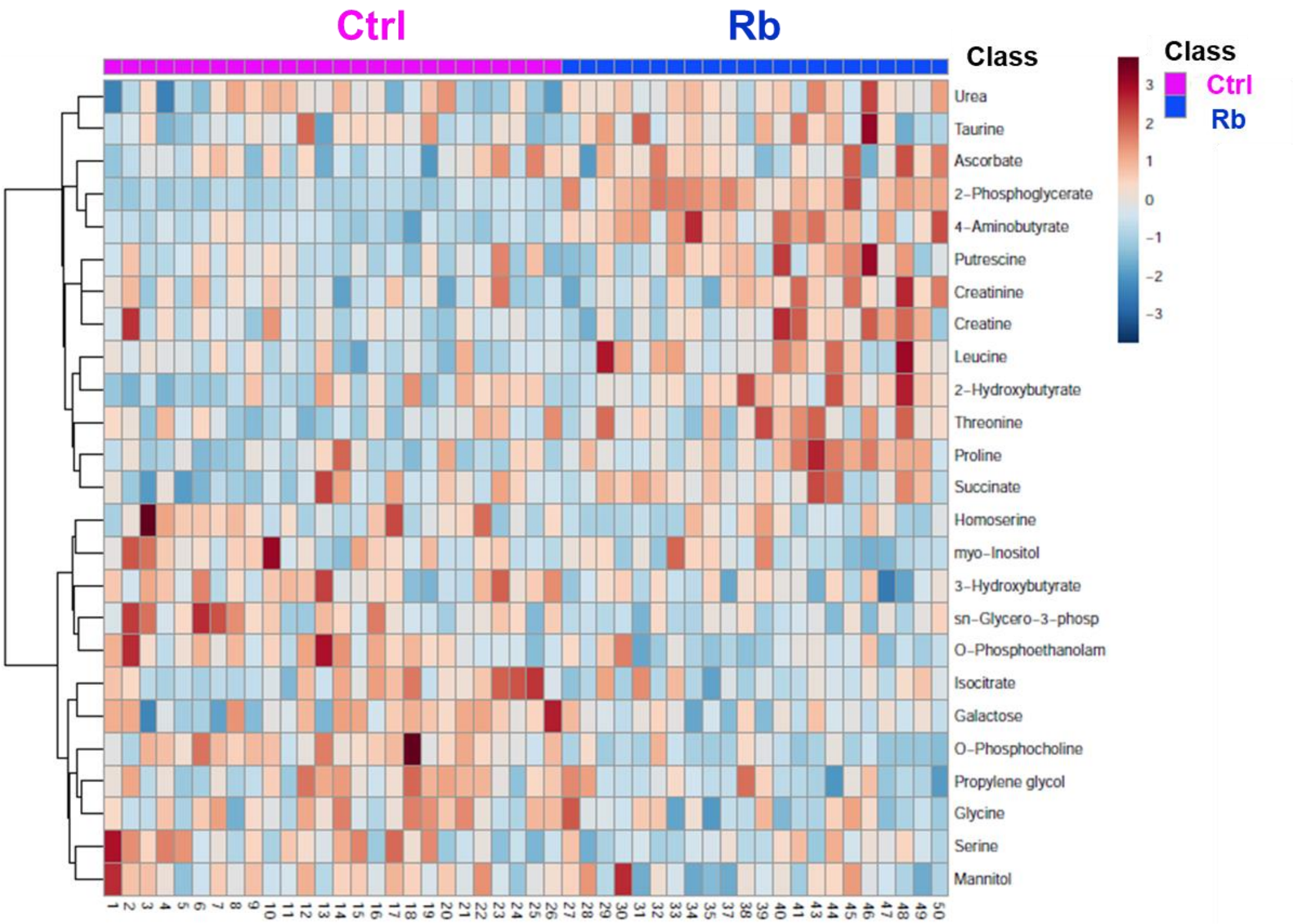

Figure S4: (A) 2D PLS-DA score plot generated for the concentration dataset of 75 metabolites annotated from ^{1}H NMR spectra of serum samples from CL3 (pink) and CG3 (red). **(B)** VIP score plot generated on the basis of PLS-DA analysis of CL3 versus CG3 depicting the top 20 significantly altered metabolites among different sub-groups. **(C)** Bar plots representing the performance measurements: accuracy, goodness of fit (R^2), and predictability (Q^2) up to the 5th component of PLS-DA model attained following the 10-fold cross validation analysis of multivariate data. The best classifier of the model is marked using the red star symbol. **(D)** Cross validation performance measurement values are displayed for all the five components of the PLS-DA model.

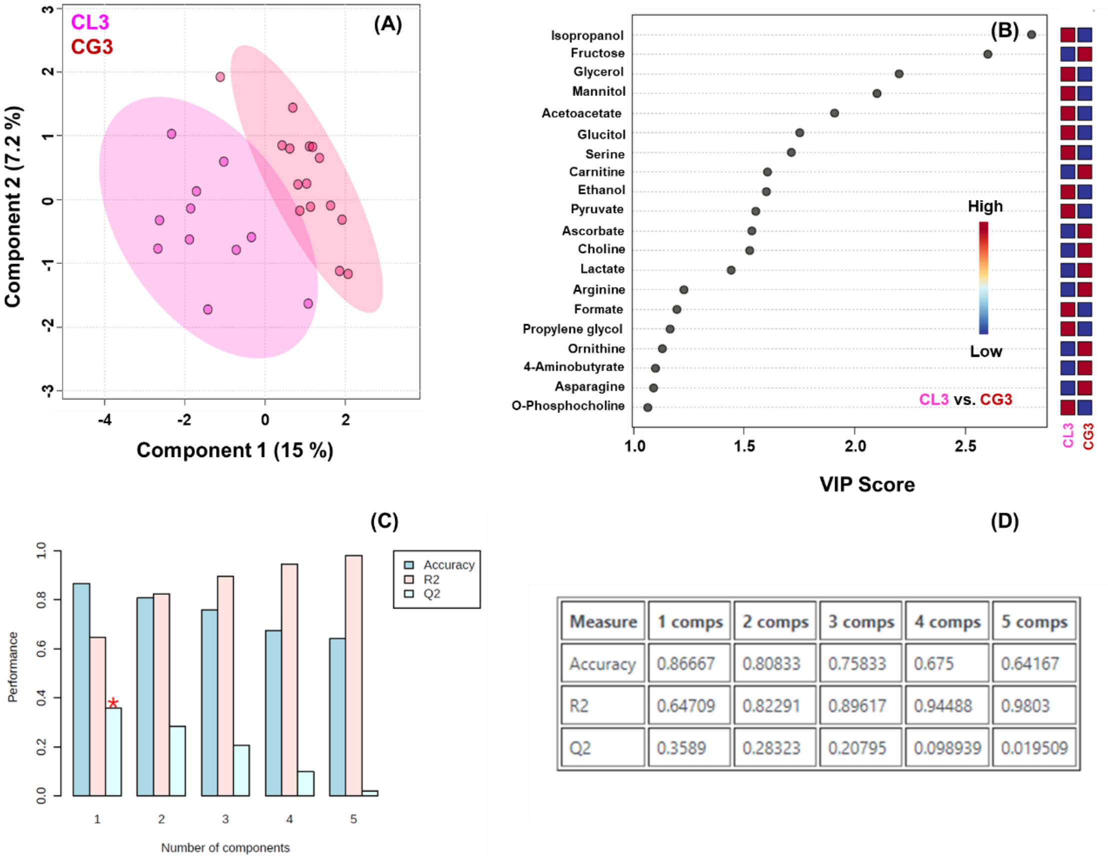

Measure	1 comps	2 comps	3 comps	4 comps	5 comps
Accuracy	0.86667	0.80833	0.75833	0.675	0.64167
R2	0.64709	0.82291	0.89617	0.94488	0.9803
Q2	0.3589	0.28323	0.20795	0.098939	0.019509

Figure S5: Bar plots representing the performance measurements (accuracy, goodness of fit (R^2) and predictability (Q^2) up to the 5th component of PLS-DA model generated for the concentration dataset of 72 metabolites from the serum samples of **(A)** CL3 versus RL3, **(C)** CL3 versus RL3U, **(E)** CL3 versus RL3B, using the 10-fold cross validation analysis. The best classifier of the model is marked using the red star symbol. Cross validation performance measurement values are displayed for all the five components of the PLS-DA model for **(B)** CL3 versus RL3, **(D)** CL3 versus RL3U, **(F)** CL3 versus RL3B. A 10-fold cross validation of the best PLS-DA model for CL3 vs. RL3 displayed the accuracy value of 0.9, R^2= 0.74 and Q^2=0.56, for CL3 vs. RL3U: accuracy value=0.91, R^2= 0.99, Q^2= 0.75 and for CL3 vs. RL3B: accuracy value=0.95, R^2= 0.98, Q^2= 0.58.

(A)

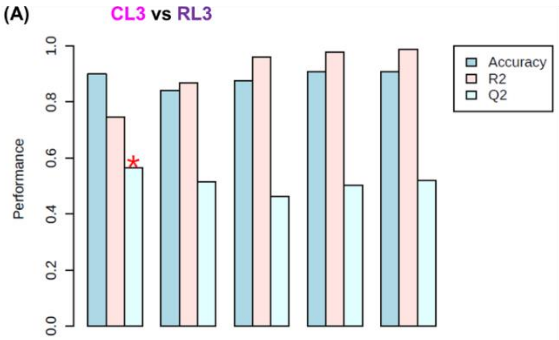

(B)

Measure	1 comps	2 comps	3 comps	4 comps	5 comps
Accuracy	0.9	0.84167	0.875	0.90833	0.90833
R2	0.74629	0.86863	0.96012	0.97856	0.98827
Q2	0.56506	0.51622	0.46301	0.5027	0.5205

(C)

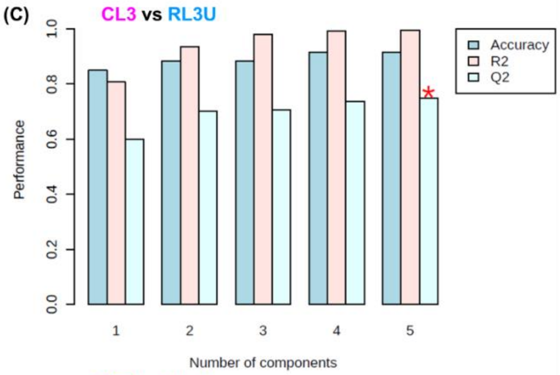

(D)

Measure	1 comps	2 comps	3 comps	4 comps	5 comps
Accuracy	0.85	0.88333	0.88333	0.91667	0.91667
R2	0.80764	0.93524	0.97953	0.99266	0.99631
Q2	0.60085	0.70255	0.70775	0.73645	0.75001

(E)

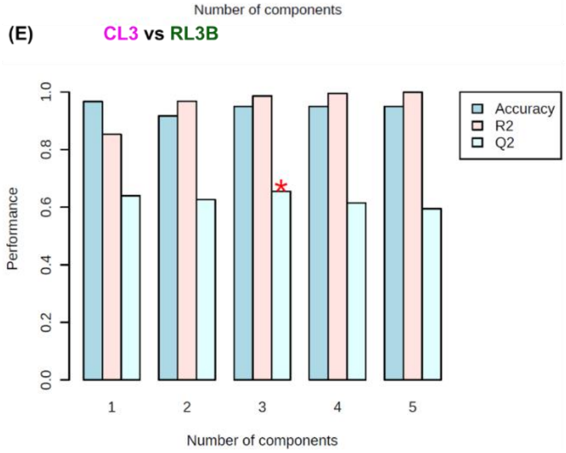

(F)

Measure	1 comps	2 comps	3 comps	4 comps	5 comps
Accuracy	0.9	0.9	0.95	0.95	0.95
R2	0.83597	0.9645	0.9831	0.99379	0.99789
Q2	0.59949	0.55273	0.58865	0.56352	0.53157

Figure S6: Heat map displaying the z-scores of top 25 discriminatory metabolites altered in RL3 with respect to CL3. X-axis represents the serum samples from CL3 (CL3; lanes:1-11; pink) and RL3 (RL3; lanes: 12-31; purple). The color gradients signify the differential metabolite concentration in the serum samples, with dark red and dark blue implies the highest and lowest metabolite concentrations.

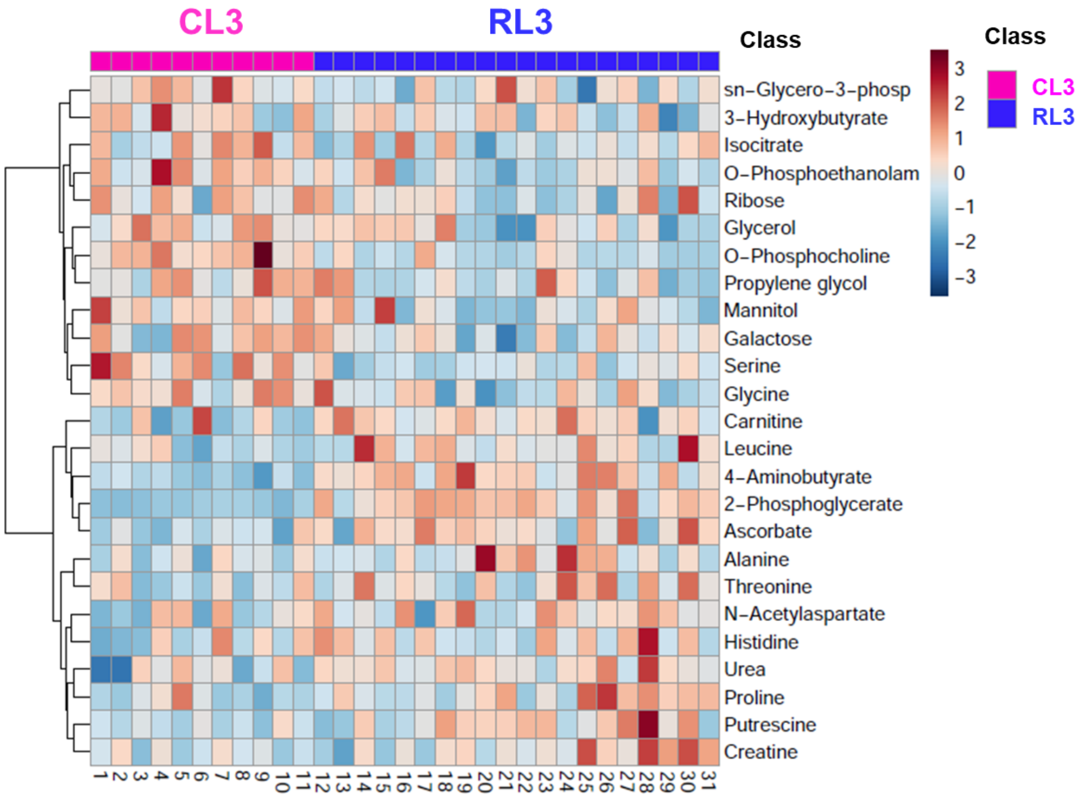

Figure S7: Heat map displaying the z-scores of top 25 discriminatory metabolites altered in RL3U with respect to CL3. X-axis represents the serum samples from CL3 (CL3; lanes:1-11; pink) and RL3U (RL3U; lanes: 12-24; blue). The color gradients signify the differential metabolite concentration in the serum samples, with dark red and dark blue implies the highest and lowest metabolite concentrations.

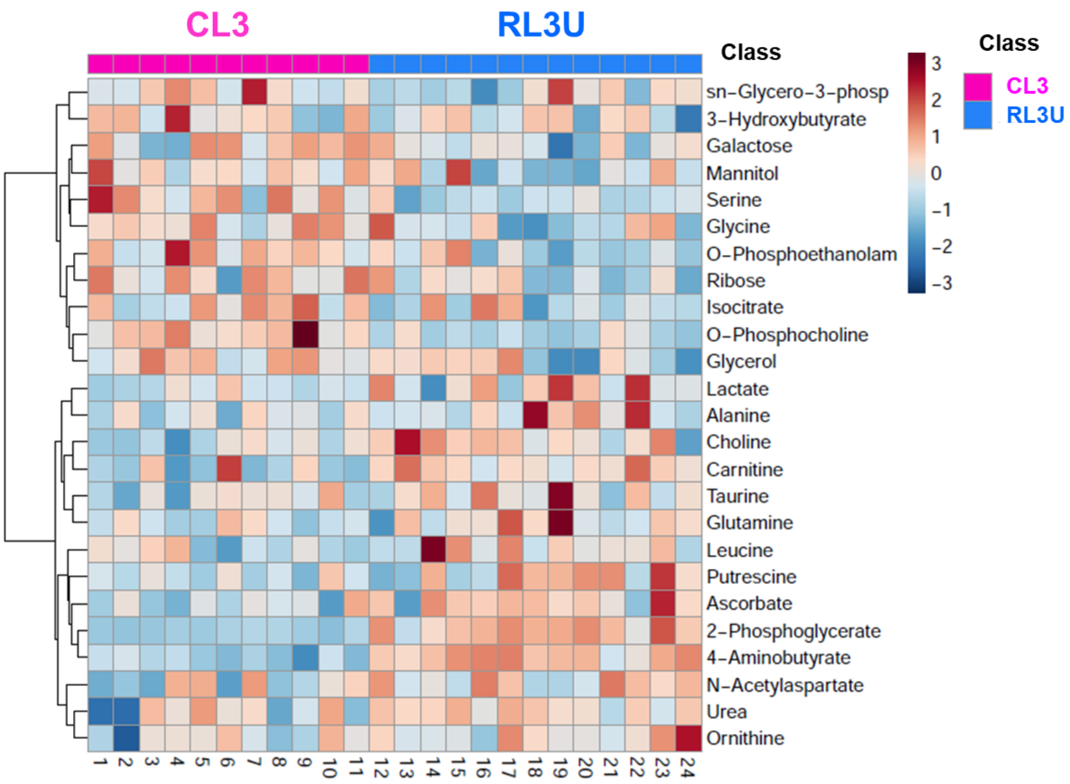

Figure S8: Heat map displaying the z-scores of top 25 discriminatory metabolites altered in RL3B with respect to CL3. X-axis represents the serum samples from CL3 (CL3; lanes: 1-11; pink) and RL3B (RL3B; lanes: 12-18); blue). The color gradients signify the differential metabolite concentration in the serum samples, with dark red and dark blue implies the highest and lowest metabolite concentrations.

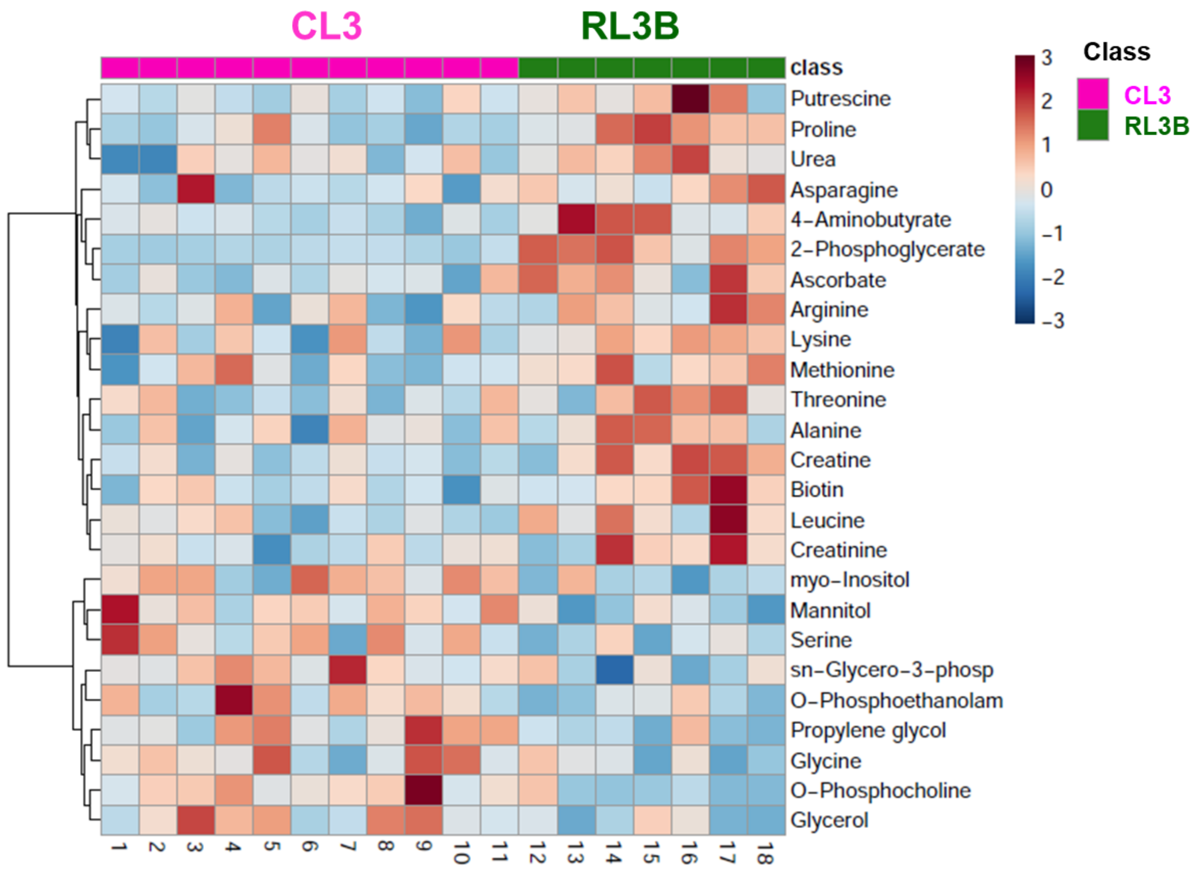

Figure S9: (A) 2D PLS-DA score plot generated for the concentration dataset of 75 metabolites annotated from ^{1}H NMR spectra of serum samples from CL3 (CL3, pink), RL3U (blue), RL3B (green). **(B)** VIP score plot generated on the basis of PLS-DA analysis of CL3 versus RL3U and RL3B depicting the top 20 significantly altered metabolites among different sub-groups. **(C)** Bar plots representing the performance measurements (accuracy, goodness of fit (R^2) and predictability (Q^2)) up to the 5th component of PLS-DA model attained following the 10-fold cross validation analysis of multivariate data. The best classifier of the model is marked using the red star symbol. **(D)** Cross validation performance measurement values are displayed for all the five components of the PLS-DA model. Cross validation parameters for the best PLS-DA model include the accuracy, R^2, and Q^2 equals to 0. 59, 0.75, and 0.56 respectively.

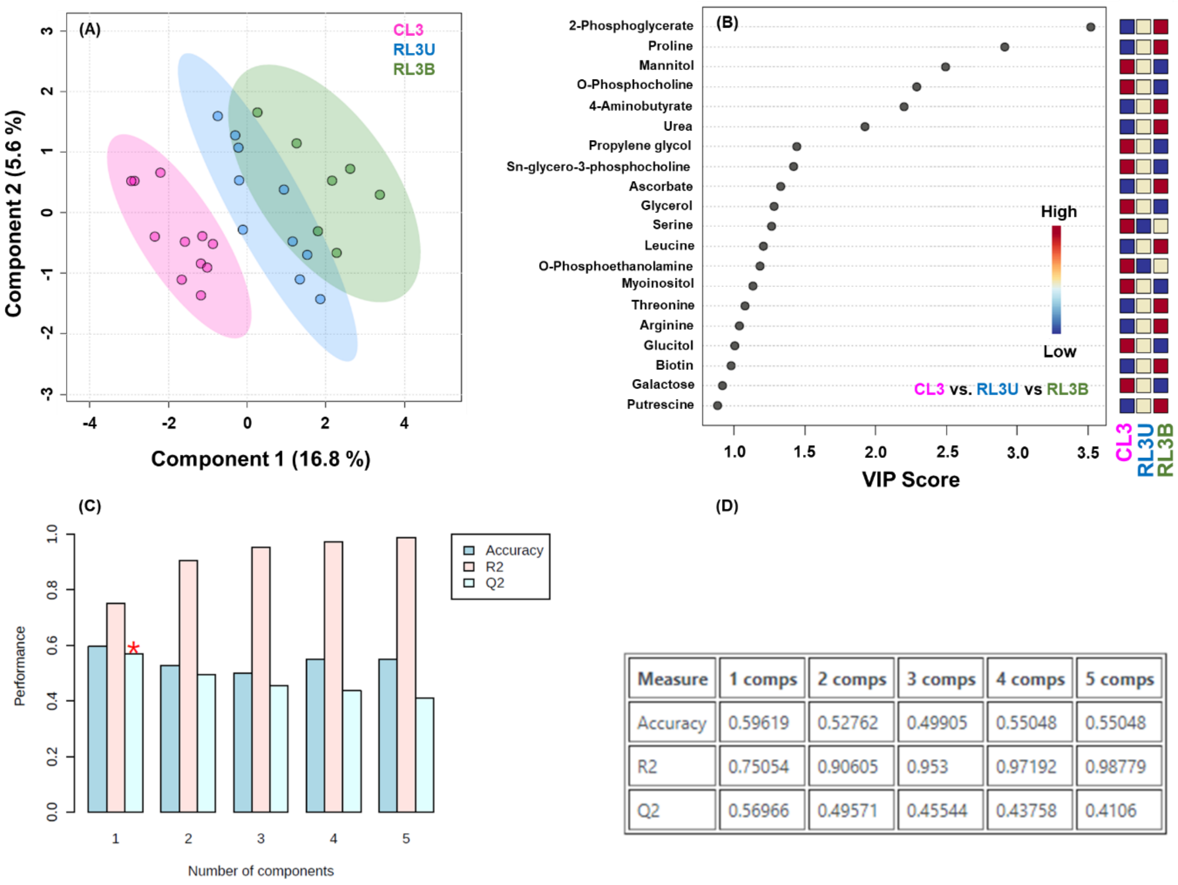

Measure	1 comps	2 comps	3 comps	4 comps	5 comps
Accuracy	0.59619	0.52762	0.49905	0.55048	0.55048
R2	0.75054	0.90605	0.953	0.97192	0.98779
Q2	0.56966	0.49571	0.45544	0.43758	0.4106

Figure S10: (A) 2D PLS-DA score plot generated for the concentration dataset of 75 metabolites annotated from ^{1}H NMR spectra of serum samples from healthy CG3 (red), with RG3U (yellow). **(B)** VIP score plot generated on the basis of PLS-DA analysis of CG3 versus RG3U depicting the top 20 significantly altered metabolites among different sub-groups. **(C)** Bar plots representing the performance measurements (accuracy, goodness of fit (R^2) and predictability (Q^2)) up to the 5th component of PLS-DA model attained following the 10-fold cross validation analysis of multivariate data. The best classifier of the model is marked using the red star symbol. **(D)** Cross validation performance measurement values are displayed for all the five components of the PLS-DA model. Cross validation parameters of the best PLS-DA model showed accuracy, R^2, and Q^2 values equal to 0. 96, 0.98, and 0.64 respectively.

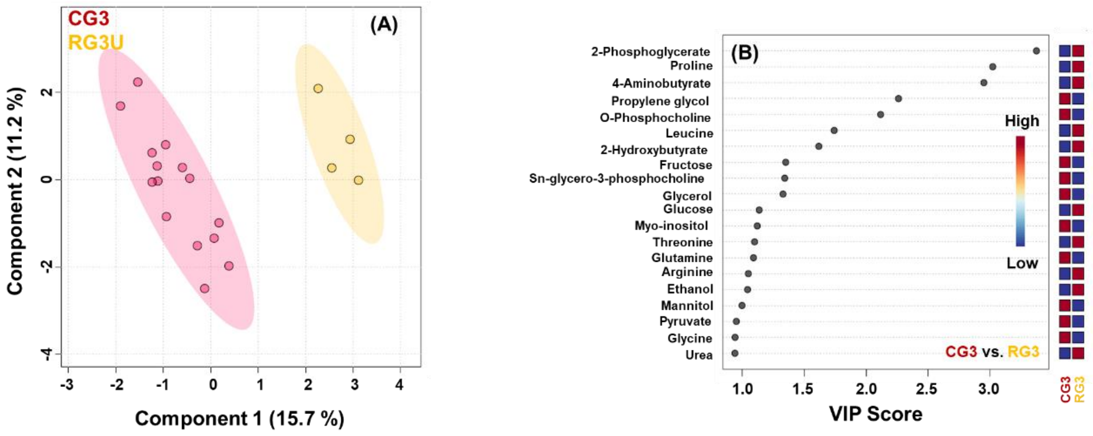

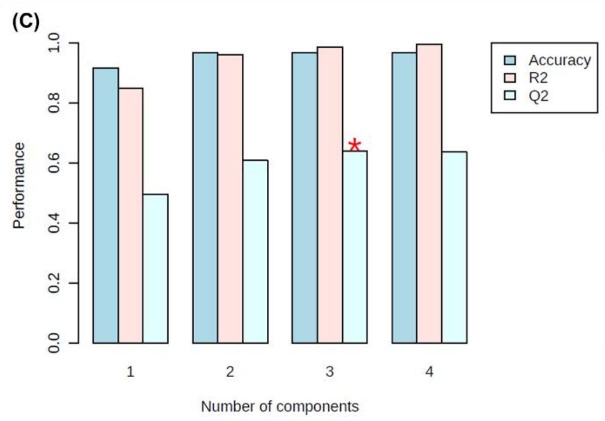

(D)

Measure	1 comps	2 comps	3 comps	4 comps
Accuracy	0.91667	0.96667	0.96667	0.96667
R2	0.84737	0.95945	0.98603	0.99582
Q2	0.49481	0.60818	0.64042	0.63609

Figure S11: The fitting of 2-hydroxybutyrate and 4-Aminobutyrate peaks in the Chenomx are shown below. The NMR signals corresponding to 2-hydroxybutyrate and 4-aminobutyrate were identified and assigned by matching their specific chemical shifts and peak patterns using Chenomx software. Blue and red peak lines indicate the fitting of metabolites, and the black lines display experimental peak patterns.

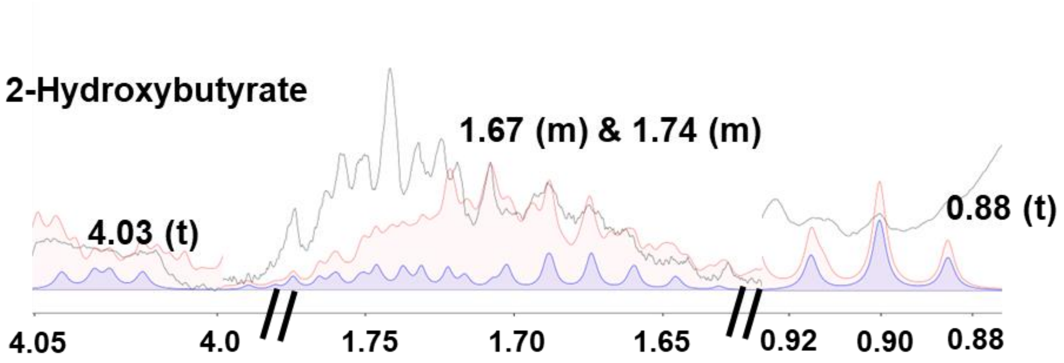

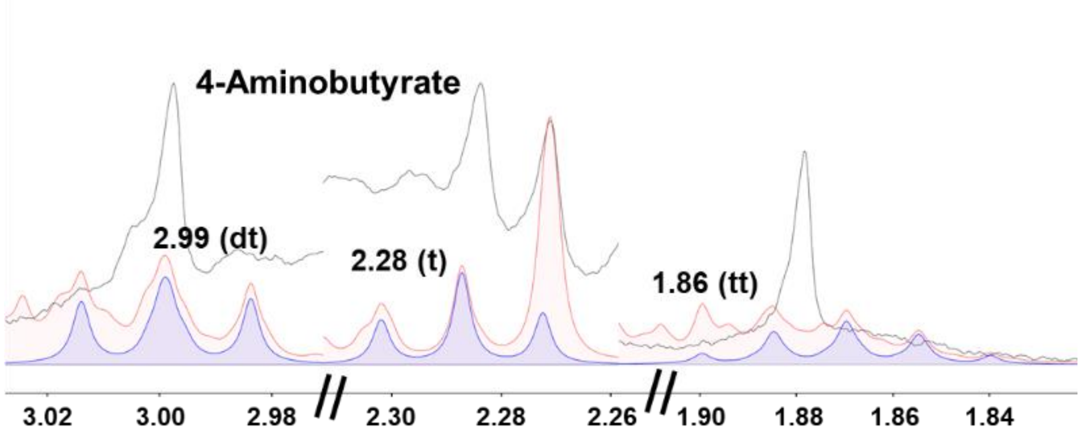

Table S1: Clinical details of Rb patients and controls. (F/M: female/male), (Age <=3: CL3/RL3, Age > 3: CG3/RG3)

**Sample**	**Sex (F/M)**	**Age (years)**	**Category (RL3 /RG3)**	**Laterality (Unilateral/Bilateral)**
Rb1	F	3	RL3	Unilateral
Rb2	M	3	RL3	Unilateral
Rb3	M	3	RL3	Unilateral
Rb4	M	3	RL3	Unilateral
Rb5	M	3	RL3	Unilateral
Rb6	M	2	RL3	Bilateral
Rb7	M	3 months	RL3	Unilateral
Rb8	F	3 months	RL3	Bilateral
Rb9	M	3	RL3	Unilateral
Rb10	F	2	RL3	Unilateral
Rb11	F	2	RL3	Unilateral
Rb12	F	1	RL3	Unilateral
Rb13	M	11 months	RL3	Unilateral
Rb14	F	6	RG3	Unilateral
Rb15	F	4	RG3	Unilateral
Rb16	F	1	RL3	Bilateral
Rb17	F	1	RL3	Bilateral
Rb18	M	5	RG3	Unilateral
Rb19	M	3	RL3	Unilateral
Rb20	M	2 months	RL3	Bilateral
Rb21	F	5 months	RL3	Unilateral
Rb22	M	1	RL3	Bilateral
Rb23	M	5 months	RL3	Bilateral
Rb24	M	4	RG3	Unilateral

C1	M	1	CL3	NA
C2	M	8	CG3	NA
C3	F	5	CG3	NA
C4	M	6 months	CL3	NA
C5	F	5	CG3	NA
C6	F	9	CG3	NA
C7	M	9	CG3	NA
C8	M	7	CG3	NA
C9	M	3	CL3	NA
C10	F	8	CG3	NA
C11	F	8	CG3	NA
C12	M	9	CG3	NA
C13	M	2	CL3	NA
C14	M	0	CL3	NA
C15	M	1	CL3	NA
C16	F	1	CL3	NA
C17	M	1	CL3	NA
C18	F	1	CL3	NA
C19	F	1	CL3	NA
C20	M	7	CG3	NA
C21	M	8	CG3	NA
C22	M	3	CL3	NA
C23	M	9	CG3	NA
C24	M	9	CG3	NA
C25	M	4	CG3	NA
C26	F	9	CG3	NA

Table S2: List of metabolites identified from 1D ^{1}H-NMR spectra attained for serum samples from Rb patients and controls (peak types: s = singlet, d= doublet, t= triplet, q= quartet, m=multiplet, td =triplet of doublet, tt = triplet of triplet, dd=doublet of doublet, ddd = doublet of doublet of doublet, quint = quintet, dtd = doublet of triplet of doublet). The fitting of 2-hydroxybutyrate and 4-Aminobutyrate peaks in the Chenomx are presented in Figure S11.

	**Compounds**	**Assignment**	**Chemical shift (δ) in ppm**
**Amino acids and their Derivatives**			
1.	Alanine	C1H3<br>C2H1	1.46 (d)<br>3.77 (q)
2.	Arginine	N9H<br>N11H, N12H2<br>C8H1<br>C5H2<br>C7H2<br>C6H2	7.22 (s)<br>6.66 (s)<br>3.76 (t)<br>3.23 (t)<br>1.92/1.88 (m)<br>1.71/1.63 (m)
3.	Asparagine	C4H2<br>C5H<br>N1H2<br>N6H2	2.84/2.94 (dd)<br>3.98 (t)<br>6.90 (s)<br>7.62 (s)
4.	Aspartate	C2H<br>C3H2	3.89 (t)<br>2.80 & 2.66 (dd)
5.	Cysteine	C2H<br>C3H2	3.97 (dd)<br>3.09 / 3.02 (m)
6.	Glycine	C2H2	3.56 (s)
7.	Histidine	C7H1<br>C5H1<br>C2H1<br>C3H2	7.86 (d)<br>7.08 (s)<br>3.98 (dd)<br>3.23 (dd)& 3.13(dd)
8.	Homoserine	C2H1<br>C4H1<br>C3H2	3.84 (t)<br>3.77 (dt)<br>2.12(dtd)&2.01(dtd)
9.	Leucine	C5H1<br>C2H3<br>C4H3<br>C1H3, C3H3	3.72 (q)<br>1.70 (m)<br>1.69 (m)<br>0.95 (t), 0.94 (t)
10.	Lysine	C6H1<br>C2H2<br>C5H1<br>C3H2<br>C4H2	3.74 (t)<br>3.02 (t)<br>1.91/1.86 (dtd)<br>1.71 (tt)<br>1.49/1.42 (m)
11.	N-Acetylaspartate	N4H1<br>C5H1	7.91 (d)<br>4.38 (m)

		C6H2<br>C1H3	2.68 (dd)/ 2.48(dd)<br>2.0 (s)
12.	Phenylalanine	C5H1 & C7H1<br>C6H1<br>C4H1 & C8H1<br>C2H1<br>C3H1 & C9H1	7.42 (m)<br>7.36 (m)<br>7.32 (m)<br>3.99 (m)<br>3.27 (m)/ 3.11 (m)
13.	Proline	C4H1<br>C7H2<br>C3H2<br>C5H2<br>C6H2	4.08 (dd)<br>3.37 (dt) /3.41 (dt)<br>2.19 (m)<br>2.00 (m)/ 1.97 (m)
14.	Serine	C3H2<br>C2H1	3.98 (m) / 3.93 (M)<br>3.83 (dd)
15.	Threonine	C2H1<br>C4H1<br>C1H3	4.25 (m)<br>3.58 (d)<br>1.31 (d)
16.	Tryptophan	NH<br>C11H<br>C8H<br>C5H<br>C9H1<br>C10H1<br>C2H1<br>C3H2<br>C3H2	10.17 (d)<br>7.72 (m)<br>7.53 (d)<br>7.31 (s)<br>7.27 (m)<br>7.19 (m)<br>4.05 (dd)<br>3.47 (dd)<br>3.29 (dd)
17.	Tyrosine	C5H1 & C10H1<br>C6H1 & C9H1<br>C2H1<br>C3H2	7.18 (ddd)<br>6.89 (ddd)<br>3.93 (t)<br>3.11 (dd)&3.04 (dd)
18.	Betaine	C5H2<br>C1H3&C3H3& C4H3	3.88 (s)<br>3.25 (s)
19.	Carnitine	C3H<br>C2H2<br>N5(CH3)3<br>C4H2	4.56 (m)<br>3.43 (m) & 3.39 (m)<br>3.21 (t)<br>2.44 (q) & 2.40 (q)
20.	Choline	C2H2<br>C3H2<br>C5H3&C6H3& C7H3	4.05 (m)<br>3.50 (m)<br>3.18 (t)
21.	Creatine	C3H2<br>C1H3	3.92 (s)<br>3.02 (s)
22.	Creatinine	C3H<br>C1H	4.04(S)<br>3.02(s)
23.	Glutamate	C2H<br>C4H2	3.75 (t)<br>2.36 (m) & 2.32 (m)

		C3H2	2.10 (td)& 2.04(td)
24.	Glutamine	N1H2<br>N7H2<br>C6H1<br>C7H2<br>C5H2	7.58 (s)<br>6.87 (s)<br>3.76 (t)<br>2.46 / 2.42 (m)<br>2.12 /2.07 (dt)
25.	Glutathione	N13H1<br>N7H1<br>C11H1<br>C2H3<br>C11H<br>C9H2<br>C4H2<br>C3H2	8.49 (d)<br>8.24 (q)<br>4.56 (m)<br>3.78 / 3.77 (m)<br>3.75 (q)<br>2.97 / 2.92 (dd)<br>2.54 / 2.52 (m)<br>2.16 / 2.12 (m)
26.	Isoleucine	C5H2<br>C3H1<br>C2H2<br>C4H2<br>C1H3	3.66 (d)<br>1.96 (m)<br>1.46/1.24 (m)<br>1.00 (d)<br>0.92 (t)
27.	Ornithine	C5H1<br>C2H2<br>C4H2<br>C2H2<br>C3H2	3.77 (m)<br>3.05 (m)<br>1.93 (m)<br>1.81 (m)<br>1.73 (m)
28.	Methionine	C5H<br>C3H2<br>C2H3<br>C1H3 & C4H2	3.85 (dd)<br>2.63 (t)<br>2.19 (s)<br>2.11 (m) & 2.19 (m)
29.	Urea	N1H2 & N3H2	5.76 (s)
30.	Valine	C2H2<br>C3H<br>C4H3<br>C4'H3	3.60 (d)<br>2.26 (m)<br>1.02 (d)<br>0.97 (d)
**Organic Acids**			
31.	2-Hydroxybutyrate	C1H3<br>C2H2<br>C3H1	0.88 (t)<br>1.67 (m) & 1.74 (m)<br>4.03 (t)
32.	2-Phosphoglycerate	C5H2<br>C4H1	3.80 (d) & 3.90 (d)<br>4.46 (dt)
33.	3-Hydroxybutyrate	C2H<br>C4H2<br>C1H3	4.14 (td)<br>2.39 (dd)& 2.29(dd)

			1.18 (d)
34.	4-Aminobutyrate	C3H2<br>C4H2<br>C2H2	1.88 (tt)<br>2.28 (t)<br>3.00 (dt)
35.	Acetate	C1H3	1.90 (m)
36.	Acetoacetate	C4H2<br>C1H3	3.43 (s)<br>2.26 (S)
37.	Ascorbate	C7H2<br>C5H<br>C4H	3.71 (d)/3.75 (d)<br>4.01 (td)<br>4.50 (d)
38.	Biotin	N15H1<br>N12H1<br>C11H1<br>C16H1<br>C12H1<br>C10H2<br>C4H2<br>C7H2<br>C5H2<br>C6H2	6.49 (m)<br>6.36 (m)<br>4.60 (m)<br>4.43 (ddd)<br>3.34 (td)<br>2.88 (dd), 2.77(dd)<br>2.20 (t) & 2.17 (t)<br>1.65 (td)<br>1.59 (tt) &1.58(tt)<br>1.40 (m)
39.	Caprate	C9H2<br>C8H2<br>C7H2<br>C6H2<br>C5H2<br>C4H2<br>C3H2<br>C2H2<br>C1H3	2.15 (t)<br>1.52 (tt)<br>1.27 (m)<br>1.26 (m)<br>1.26 (tt)<br>1.25 (m)<br>1.25 (m)<br>1.24 (m)<br>0.84 (t)
40.	Citrate	C2H2 & C5H2	2.68 (d) & 2.52 (d)
41.	Pyruvate	C1H3	2.36 (s)
42.	Succinate	C4H2, C5H2	2.38 (s)
43.	Formate	C2H1	8.41 (s)
44.	Lactate	C2H1<br>C1H3	4.10 (q)<br>1.31 (d)
45.	Glucuronate	C4H1<br>C6H1<br>C10H2<br>C12H1<br>C8H1	5.23 (d)/ 4.07 (d)<br>4.63 (dd)<br>3.71 (m)/3.60 (m)<br>3.50 (dd)<br>3.26 (t)
46.	Malate	C5H1<br>C4H2	4.30 (dd)<br>2.66 (dd)/2.36 (dd)
47.	Malonate	C4H2	3.10 (s)

**Carbohydrates**			
48.	myo-Inositol	C5H1<br>C1H/C3H<br>C4H/C6H<br>C2H	4.05 (t)<br>3.61 (dd)<br>3.52 (dd)<br>3.25 (t)
49.	Fructose	C11H1<br>C9H1<br>C3H1<br>C2H2<br>C7H2	4.11 (dd)<br>4.9 (s)<br>4.03 (dt)<br>3.87 (m)<br>3.67 (d) & 3.64 (d)
50.	Galactose	C5H<br>C11H<br>C7H<br>C2H<br>C9H<br>C3H	5.25 (d)/4.57 (d)<br>3.99 (d)<br>3.81 (dd)<br>3.75 (quint)<br>3.64 (dd)<br>3.71(m)/4.07 (m)
51.	Glucitol	C5H 1<br>C3H1<br>C9H1<br>C7H1	3.84 (dd)<br>3.83 (td)/ 3.82(td)<br>3.83 (dd)& 3.64(dd)<br>3.64 (dd)/ 3.61(dd)
52.	Glucose	C5H1<br>C2H2<br>C3H<br>C9H1<br>C11H1<br>C3H1	5.22 (d)/ 4.93 (d)<br>3.89 (quint)/ 3.80 (quint)<br>3.59 (dd)<br>3.39 (dd)<br>3.45 (dd)
53.	Glycerol	C3H1<br>C5H2 & C2H2	3.77 (m)<br>3.54 (dd)& 3.64(dd)
54.	Ribose	C2H1<br>C3H1<br>C9H1<br>C7H1<br>C5H1	3.60 (m)<br>3.85 (m)<br>4.0(m)<br>3.51(m)<br>5.24 (m)
55.	Propylene glycol	C2H1<br>C4H2<br>C1H3	3.87 (m)<br>3.54 (dd)/ 3.48(dd)<br>1.13 (d)
56.	Mannitol	C2H2/C11H2<br>C3H/C9H<br>C5H/C3H<br>C11H2	3.86 (dd)/ 367 (q)<br>3.74 (m)<br>3.78 (d)<br>3.67 (dd)
**Nucleotides**			
57.	dTTP	C3H1<br>C5H1<br>C7H1<br>C9H1	7.68 (d)<br>6.32 (m)<br>4.73 (m)<br>4.61 (m)

		C10H1<br>C6H2<br>C1H3	4.18 (m)<br>2.38(m)<br>1.91(d)
58.	Hypoxanthine	C4H1<br>C8H1	8.19 (S)<br>8.17 (S)
**Others**			
59.	Putrescine	C5H2, C2H2<br>C3H2, C4H2	3.04 (t)<br>1.75 (m)
60.	Dimethyl sulfone	C1H3 & C3H3	3.1 (s)
61.	Ethanol	C2H2<br>C1H3	3.64 (q)<br>1.15 (t)
62.	Histamine	C7H1<br>C4H1<br>C2H2<br>C3H2	7.82 (s)<br>7.10 (m)<br>3.28 (t)<br>2.99 (m)
63.	Taurine	C2H2<br>C3H2	3.41(t)<br>3.24 (m)
64.	Melatonin	N12H1<br>C5H1<br>C8H1<br>C17H1<br>C4H1<br>C1H3<br>C11H2<br>C10H2<br>C14H1	7.9 (t)<br>7.42 (dd)<br>7.21 (d)<br>7.20 (s)<br>6.91 (d)<br>3.88 (S)<br>3.47 (q)<br>2.93 (t)<br>1.89 (s)
65.	Methanol	C1H3	3.35 (s)
66.	O-Phosphocholine	C6H2<br>C5H1<br>C1H3/C3H3/C4H3	4.15 (dt)<br>3.57 (dt)<br>3.20 (s)
67.	O-Phosphoethanolamine	C3H2<br>C2H2	3.95 (m)<br>3.21 (t)
68.	Isopropanol	C2H3<br>C3H3 & C1H3	4.014 (m)<br>1.15 (d)
69.	Isocitrate	C9H1<br>C5H1<br>C2H1	4.05 (d)<br>2.98 (td)<br>2.52 / 2.46 (dd)
70.	Acetoin	C1H3<br>C4H1<br>C5H3	2.21 (s)<br>4.42 (q)<br>1.36 (d)
71.	Acetone	C1H6	2.22 (s)
72.	sn-Glycero-3-phosphocholine	C6H2<br>C12H3<br>C5H4<br>C1H9	4.31 (m)<br>3.90 (m)<br>3.67 (m)<br>3.20 (s)

Table S3: Comparative analysis of significantly altered metabolic pathways with -log 10(p) values > 0.5 and pathway impact values > 0.01 obtained via pairwise pathway impact analysis of all the Rb patients with respect to controls.

S. No.	Altered Pathways	-Log 10(p)	Impact
1	Glycerophospholipid metabolism	6.81	0.11
2	Butanoate metabolism	5.99	0.14
3	Arginine and proline metabolism	5.80	0.48
4	Alanine, aspartate, and glutamate metabolism	4.57	0.71
5	Sphingolipid metabolism	2.62	0.01
6	Purine metabolism	1.68	0.02
7	Primary bile acid biosynthesis	1.55	0.02
8	Phosphatidylinositol signaling system	1.32	0.04
9	Arginine biosynthesis	1.19	0.25
10	Citrate cycle (TCA cycle)	1.06	0.26
11	Glycine, serine, and threonine metabolism	0.99	0.30
12	Synthesis and degradation of ketone bodies	0.93	0.60
13	Taurine and hypotaurine metabolism	0.87	0.43
14	Glutathione metabolism	0.65	0.37
15	Ascorbate and aldarate metabolism	0.65	0.50
16	Inositol phosphate metabolism	0.65	0.13
17	Galactose metabolism	0.57	0.05
18	Glyoxylate and dicarboxylate metabolism	0.52	0.14

Table S4: Comparative analysis of top- most metabolites with VIP score greater than 1 based on PLS-DA model generated for different subgroups including: CL3 vs RL3, CL3 vs. RL3U, CL3 vs. RL3B. Green and red color denotes increased and decreased levels of metabolites in serum from Rb patients in contrast to controls.

S. No.	CL3 vs. RL3	CL3 vs. RL3U	CL3 vs. RL3B
1	O-Phosphocholine ↓	O-Phosphocholine ↓	O-Phosphocholine ↓
2	2-Phosphoglycerate ↑	2-Phosphoglycerate ↑	2-Phosphoglycerate ↑
3	4-Aminobutyrate ↑	4-Aminobutyrate ↑	4-Aminobutyrate ↑
4	Proline ↑	Proline ↑	Proline ↑
5	O-Phosphoethanolamine ↓	O-Phosphoethanolamine ↓	O-Phosphoethanolamine ↓
6	Urea ↑	Urea ↑	Urea ↑
7	Leucine ↑	Leucine ↑	Leucine ↑
8	Ascorbate ↑	Ascorbate ↑	Ascorbate ↑
9	Glycerol ↓	Glycerol ↓	Glycerol ↓
10	Serine ↓	Serine ↓	Serine ↓
11	Mannitol ↓	Mannitol ↓	Mannitol ↓
12	Carnitine ↑	Carnitine ↑	-
13	Isocitrate ↓	Isocitrate ↓	-
14	Glycine ↓	Glycine ↓	-
15	Ribose ↓	Ribose ↓	-
16	Galactose ↓	Galactose ↓	-
17	-	Lactate ↑	-
18	-	Glucuronate ↑	-
19	-	Choline ↑	-
20	Sn-Glycero-3-phosphocholine ↓	-	Sn-Glycero-3-phosphocholine ↓
21	Propylene glycol ↓	-	Propylene glycol ↓
22	Arginine ↑		Arginine ↑
23	Glucitol ↓	-	Glucitol ↓
24	-	-	Threonine ↑
25	-	-	Myoinositol ↑
26	-	-	Biotin ↑
27	-	-	Acetoacetate ↑
28	-	-	Putrescine ↑

Table S5: Pairwise pathway impact analysis of CL3 versus RL3 depicting the significantly altered metabolic pathways along with their -log 10(p) values and pathway impact values calculated based on the pathway enrichment and pathway topology analysis respectively.

S. No.	Altered Metabolic pathways	-Log 10(p)	Impact
1	Glycerophospholipid metabolism	5.199	0.11
2	Arginine and proline metabolism	3.198	0.48
3	Alanine, aspartate and glutamate metabolism	3.083	0.71
4	Butanoate metabolism	2.826	0.14
5	Sphingolipid metabolism	2.314	0.01
6	Aminoacyl-tRNA biosynthesis	1.941	0.00
7	Purine metabolism	1.916	0.02
8	Arginine biosynthesis	1.648	0.25
9	Glycerolipid metabolism	1.456	0.24
10	Ether lipid metabolism	1.442	0.00
11	Selenocompound metabolism	1.135	0.00
12	Primary bile acid biosynthesis	1.075	0.02
13	Valine, leucine, and isoleucine biosynthesis	1.069	0.00
14	Galactose metabolism	1.031	0.05
15	Glutathione metabolism	0.914	0.37
16	Glycine, serine, and threonine metabolism	0.868	0.30
17	Pentose phosphate pathway	0.849	0.00
18	Pyrimidine metabolism	0.848	0.00
19	Lysine degradation	0.802	0.00
20	Biotin metabolism	0.774	0.20
21	Glyoxylate and dicarboxylate metabolism	0.751	0.14
22	Porphyrin and chlorophyll metabolism	0.723	0.00
23	Propanoate metabolism	0.663	0.00
24	Amino sugar and nucleotide sugar metabolism	0.647	0.00
25	Citrate cycle (TCA cycle)	0.597	0.26
26	D-Glutamine and D-glutamate metabolism	0.525	0.50
27	Nitrogen metabolism	0.525	0.00
28	beta-Alanine metabolism	0.481	0.00
29	Valine, leucine, and isoleucine degradation	0.458	0.00
30	Fructose and mannose metabolism	0.451	0.03
31	Phosphatidylinositol signaling system	0.410	0.04
32	Cysteine and methionine metabolism	0.386	0.20
33	Taurine and hypotaurine metabolism	0.329	0.43
34	Synthesis and degradation of ketone bodies	0.327	0.60

35	Pyruvate metabolism	0.322	0.30
36	Glycolysis / Gluconeogenesis	0.279	0.13
37	Histidine metabolism	0.253	0.41
38	Pentose and glucuronate interconversions	0.248	0.13
39	Ascorbate and aldarate metabolism	0.241	0.50
40	Inositol phosphate metabolism	0.241	0.13
41	Tyrosine metabolism	0.206	0.14
42	Tryptophan metabolism	0.176	0.17
43	Ubiquinone and other terpenoid-quinone biosynthesis	0.170	0.00
44	Nicotinate and nicotinamide metabolism	0.120	0.00
45	Phenylalanine, tyrosine, and tryptophan biosynthesis	0.116	1.00
46	Phenylalanine metabolism	0.116	0.36
47	Fatty acid biosynthesis	0.102	0.00
48	Thiamine metabolism	0.083	0.00
49	Pantothenate and CoA biosynthesis	0.006	0.00

Table S6: Pairwise pathway impact analysis of CL3 versus RL3U depicting the significantly altered metabolic pathways along with their -log 10(p) values and pathway impact values calculated based on the pathway enrichment and pathway topology analysis respectively.

S. No.	Altered Metabolic Pathways	-Log 10(p)	Impact
1	Glycerophospholipid metabolism	4.933	0.11
2	Butanoate metabolism	3.061	0.14
3	Alanine, aspartate, and glutamate metabolism	3.045	0.71
4	Arginine and proline metabolism	2.264	0.48
5	Sphingolipid metabolism	1.937	0.01
6	Pentose phosphate pathway	1.307	0.00
7	Purine metabolism	1.278	0.02
8	Primary bile acid biosynthesis	1.177	0.02
9	Glycerolipid metabolism	1.036	0.24
10	Glycine, serine, and threonine metabolism	1.020	0.30
11	Arginine biosynthesis	0.994	0.25
12	Selenocompound metabolism	0.981	0.00
13	Ether lipid metabolism	0.918	0.00
14	Pyrimidine metabolism	0.856	0.00
15	Glutathione metabolism	0.841	0.37
16	Taurine and hypotaurine metabolism	0.709	0.43
17	Glyoxylate and dicarboxylate metabolism	0.693	0.14
18	Pentose and glucuronate interconversions	0.653	0.13
19	Pyruvate metabolism	0.620	0.30
20	Porphyrin and chlorophyll metabolism	0.620	0.00
21	Galactose metabolism	0.607	0.05
22	Amino sugar and nucleotide sugar metabolism	0.597	0.00
23	Aminoacyl-tRNA biosynthesis	0.561	0.00
24	Citrate cycle (TCA cycle)	0.545	0.26
25	Valine, leucine, and isoleucine biosynthesis	0.508	0.00
26	Ascorbate and aldarate metabolism	0.495	0.50
27	Inositol phosphate metabolism	0.495	0.13
28	D-Glutamine and D-glutamate metabolism	0.489	0.50
29	Nitrogen metabolism	0.489	0.00
30	Synthesis and degradation of ketone bodies	0.413	0.60
31	Thiamine metabolism	0.391	0.00
32	Valine, leucine, and isoleucine degradation	0.379	0.00

33	Glycolysis / Gluconeogenesis	0.359	0.13
34	beta-Alanine metabolism	0.329	0.00
35	Tryptophan metabolism	0.307	0.17
36	Propanoate metabolism	0.303	0.00
37	Tyrosine metabolism	0.301	0.14
38	Lysine degradation	0.292	0.00
39	Cysteine and methionine metabolism	0.285	0.20
40	Fructose and mannose metabolism	0.280	0.03
41	Nicotinate and nicotinamide metabolism	0.227	0.00
42	Fatty acid biosynthesis	0.153	0.00
43	Pantothenate and CoA biosynthesis	0.136	0.00
44	Biotin metabolism	0.114	0.20
45	Ubiquinone and other terpenoid-quinone biosynthesis	0.106	0.00
46	Histidine metabolism	0.094	0.41
47	Phenylalanine, tyrosine and tryptophan biosynthesis	0.031	1.00
48	Phenylalanine metabolism	0.031	0.36
49	Phosphatidylinositol signaling system	0.004	0.04

Table S7: Pairwise pathway impact analysis of CL3 versus RL3B, depicting the significantly altered metabolic pathways along with their -log 10(p) values and pathway impact values calculated based on the pathway enrichment and pathway topology analysis respectively.

S. No.	Altered Metabolic Pathways	-Log 10(p)	Impact
1	Arginine and proline metabolism	3.507	0.48
2	Aminoacyl-tRNA biosynthesis	3.369	0.00
3	Glycerophospholipid metabolism	2.501	0.11
4	Biotin metabolism	1.976	0.20
5	Galactose metabolism	1.913	0.05
6	Phosphatidylinositol signaling system	1.820	0.04
7	Butanoate metabolism	1.790	0.14
8	Ether lipid metabolism	1.640	0.00
9	Valine, leucine, and isoleucine biosynthesis	1.590	0.00
10	Glycerolipid metabolism	1.567	0.24
11	Alanine, aspartate, and glutamate metabolism	1.511	0.71
12	Purine metabolism	1.447	0.02
13	Arginine biosynthesis	1.381	0.25
14	Sphingolipid metabolism	1.330	0.01
15	Lysine degradation	1.270	0.00
16	Ascorbate and aldarate metabolism	1.002	0.50
17	Inositol phosphate metabolism	1.002	0.13
18	Selenocompound metabolism	0.965	0.00
19	Valine, leucine, and isoleucine degradation	0.963	0.00
20	Fructose and mannose metabolism	0.851	0.03
21	Glutathione metabolism	0.849	0.37
22	Ubiquinone and other terpenoid-quinone biosynthesis	0.848	0.00
23	Propanoate metabolism	0.805	0.00
24	Glycine, serine, and threonine metabolism	0.748	0.30
25	Synthesis and degradation of ketone bodies	0.657	0.60
26	Porphyrin and chlorophyll metabolism	0.609	0.00
27	Phenylalanine, tyrosine, and tryptophan biosynthesis	0.558	1.00
28	Phenylalanine metabolism	0.558	0.36
29	Pyrimidine metabolism	0.545	0.00
30	Primary bile acid biosynthesis	0.535	0.02
31	Cysteine and methionine metabolism	0.491	0.20
32	Tyrosine metabolism	0.471	0.14
33	D-Glutamine and D-glutamate metabolism	0.463	0.50
34	Nitrogen metabolism	0.463	0.00

35	beta-Alanine metabolism	0.446	0.00
36	Pentose and glucuronate interconversions	0.444	0.13
37	Thiamine metabolism	0.434	0.00
38	Histidine metabolism	0.401	0.41
39	Glyoxylate and dicarboxylate metabolism	0.339	0.14
40	Amino sugar and nucleotide sugar metabolism	0.287	0.00
41	Citrate cycle (TCA cycle)	0.285	0.26
42	Pentose phosphate pathway	0.162	0.00
43	Taurine and hypotaurine metabolism	0.148	0.43
44	Pantothenate and CoA biosynthesis	0.121	0.00
45	Glycolysis / Gluconeogenesis	0.098	0.13
46	Pyruvate metabolism	0.054	0.30
47	Nicotinate and nicotinamide metabolism	0.042	0.00
48	Tryptophan metabolism	0.008	0.17
49	Fatty acid biosynthesis	0.005	0.00

Table S8: Comparative analysis of significantly altered metabolic pathways with -log 10(p) values > 1.0 and pathway impact values > 0.01. obtained via pairwise pathway impact analysis of different subgroups CL3 vs. RL3, CL3 vs. RL3U and CL3 vs. RL3B.

S.No.	Perturbed Metabolic Pathways	CL3 vs. RL3		CL3 vs. RL3U		CL3 vs. RL3B	
		-log10(p)	Impact	-log10(p)	Impact	-log10(p)	Impact
1	Glycerophospholipid metabolism	5.20	0.11	4.93	0.11	2.50	0.11
2	Arginine and proline metabolism	3.20	0.48	2.26	0.48	3.51	0.48
3	Alanine, aspartate and glutamate metabolism	3.08	0.71	3.04	0.71	1.51	0.71
4	Butanoate metabolism	2.83	0.14	3.06	0.14	1.79	0.14
5	Sphingolipid metabolism	2.31	0.01	1.94	0.01	1.33	0.01
6	Purine metabolism	1.92	0.02	1.28	0.02	1.45	0.02
7	Arginine biosynthesis	1.65	0.25	---	---	1.38	0.25
8	Glycerolipid metabolism	1.46	0.24	1.04	0.24	1.57	0.24
9	Primary bile acid biosynthesis	1.07	0.02	1.18	0.02	---	---
10	Galactose metabolism	1.03	0.05	---	---	1.91	0.05
11	Glycine, serine, and threonine metabolism	---	---	1.02	0.30	-	-
12	Biotin metabolism	---	---	---	---	1.98	0.20
13	Phosphatidylinositol signaling system	---	---	---	---	1.82	0.04
14	Ascorbate and aldarate metabolism	---	---	---	---	1.00	0.50
15	Inositol phosphate metabolism	---	---	---	---	1.00	0.13
